# IL-13 and calpain-14 suppress the expression of SPINK7 by regulating OVOL1 in eosinophilic esophagitis

**DOI:** 10.1172/jci.insight.204687

**Published:** 2026-04-28

**Authors:** Nurit P. Azouz, Andrea M. Klingler, Sierra S. Beach, Kalen Rossey, Mark Rochman, Misu Paul, Julie M. Caldwell, Michael Brusilovsky, Alexander T. Dwyer, Xiaoting Chen, Daniel Miller, Carmy Forney, Leah C. Kottyan, Matthew T. Weirauch, Marc E. Rothenberg

**Affiliations:** 1 Division of Allergy and Immunology, Cincinnati Children’s Hospital Medical Center, Department of Pediatrics, University of Cincinnati College of Medicine, Cincinnati, Ohio, USA.; 2 Department of Pediatrics, University of Cincinnati College of Medicine, Cincinnati, Ohio, USA.; 3 Center for Autoimmune Genomics and Etiology, and; 4 Divisions of Human Genetics, Biomedical Informatics, and Developmental Biology, Cincinnati Children’s Hospital Medical Center, University of Cincinnati College of Medicine, Cincinnati, Ohio, USA.

**Keywords:** Gastroenterology, Immunology, Inflammation, Allergy, Innate immunity

## Abstract

Eosinophilic esophagitis (EoE) is a type 2 allergic disease characterized by esophageal inflammation and epithelial cell dysfunction. The acquired loss of the anti–serine protease of kazal type 7 (anti-SPINK7) in the squamous epithelium of the esophagus has a causal role in EoE pathogenesis. However, there is a limited understanding of the factors that regulate its expression and responsiveness to inflammatory stimuli. Herein, we have identified the transcription factor, ovo like transcriptional repressor 1 (OVOL1), as an esophageal selective gene product that regulates SPINK7 promoter activity. Overexpression of *OVOL1* increased *SPINK7* expression, whereas its depletion decreased *SPINK7* expression, impaired epithelial barrier, and increased production of the proatopy cytokine thymic stromal lymphopoietin (TSLP). Stimulation with IL-13 abrogated the nuclear translocation of OVOL1 and promoted enhanced degradation of OVOL1 protein. This effect of IL-13 was dependent on the esophageal specific cysteine protease calpain-14 at least in part. Analysis of human esophageal biopsies demonstrated that the expression of esophageal OVOL1 correlated with *SPINK7* transcript expression and was lost as a function of EoE disease activity. In summary, our study identifies key regulatory mechanisms in EoE pathogenesis, demonstrating that OVOL1 promotes SPINK7 transcription, whereas IL-13 suppresses this pathway in EoE.

## Abstract 2

Eosinophilic esophagitis is an allergic esophageal disease. Loss of the epithelial protein SPINK7 drives pathogenesis. We demonstrated that thetranscription factor OVOL1 promotes SPINK7 expression, while IL-13 suppresses OVOL1 nuclear localization and stability via calpain-14, exacerbating esophageal inflammation

## Graphical abstract

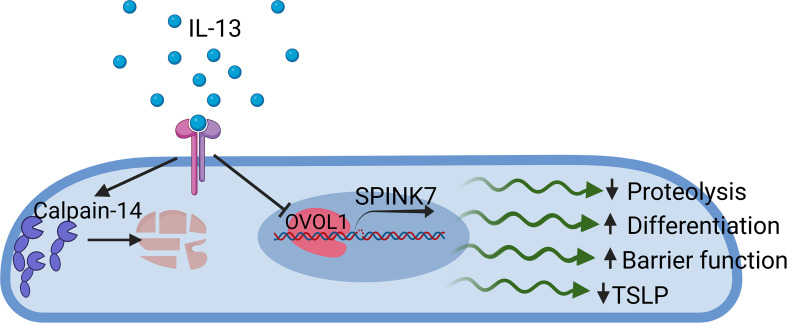

## Introduction

Eosinophilic esophagitis (EoE) is a chronic, food antigen–driven disease of the esophagus possibly afflicting over 450,000 people in the United States (1). Evidence is emerging that the primary etiology is due to dysregulated esophageal homeostasis that alters the physiological function of the esophagus as an immune-sensing organ (2–5). Mechanistically, EoE is mediated by IL-13 overexpression, as demonstrated by success of drugs that block IL-13 and/or its receptor (2, 6). The action of IL-13 includes transcriptional induction of CCL26, a key eosinophil chemoattractant and activating factor, as well as induction of calpain-14, an intracellular cysteine protease involved in esophageal barrier function, both produced by esophageal epithelial cells (7–10).

In the squamous epithelium of the skin and esophagus, expression of the anti–serine proteases of the kazal type (anti-SPINK) maintains homeostatic control of inflammation. Loss of SPINK5 and/or SPINK7 (the 2 main SPINK family members expressed in the squamous epithelium), leads to profound consequences, including impaired epithelial barrier function and elicitation of allergic inflammation in the skin and/or esophagus (11–14). Depletion of *SPINK7* in esophageal epithelial cells is sufficient to induce barrier dysfunction, activation of eosinophils by the urokinase plasminogen activator pathway, and production of proinflammatory and proatopy cytokines, including the alarmin thymic stromal lymphopoietin (TSLP) (11). Notably, variants at locus 5q22 include the TSLP gene and are associated with EoE (15, 16). Rare homozygous mutations of *SPINK5*, as well as *Spink5* deletion in mice, are sufficient to elicit loss of epithelial barrier integrity, induction of TSLP, and proinflammatory and proatopy responses including EoE in vivo (17–19). However, there are little data about how SPINKs are regulated under basal conditions, and the mechanism of their acquired loss in EoE has not yet been uncovered.

Herein, we interrogated the regulatory mechanisms that control *SPINK7* expression during epithelial differentiation. We mapped the promoter region of SPINK7 and screened for transcription factors (TFs) that may regulate this gene. We identified the C2H2 zinc finger TF, ovo like transcriptional repressor 1 (OVOL1) as an upstream regulator of *SPINK7* expression. OVOL1 is an esophageal enriched gene, which is highly expressed in the skin and the esophagus. OVOL1 is known for its regulation of epithelial differentiation and variants in the *OVOL1* gene are associated with atopic dermatitis, a type 2 allergic disease with strong similarities to EoE, including impaired barrier function (20–23). We further investigated the mechanism of action of OVOL1 in the esophageal epithelium and the effects of Th2 cytokines on OVOL1 activity.

Our findings demonstrate that OVOL1 transcriptional activity promotes esophageal epithelium homeostasis by inducing epithelial differentiation, epithelial integrity, and *SPINK7* expression. IL-13 and calpain-14 disrupt this pathway by preventing the translocation of OVOL1 to the nucleus and enhancing its degradation. Our findings identify an epithelial regulatory mechanism involved in EoE pathogenesis.

## Results

### SPINK7 expression is induced during epithelial differentiation.

**Figure 1:**
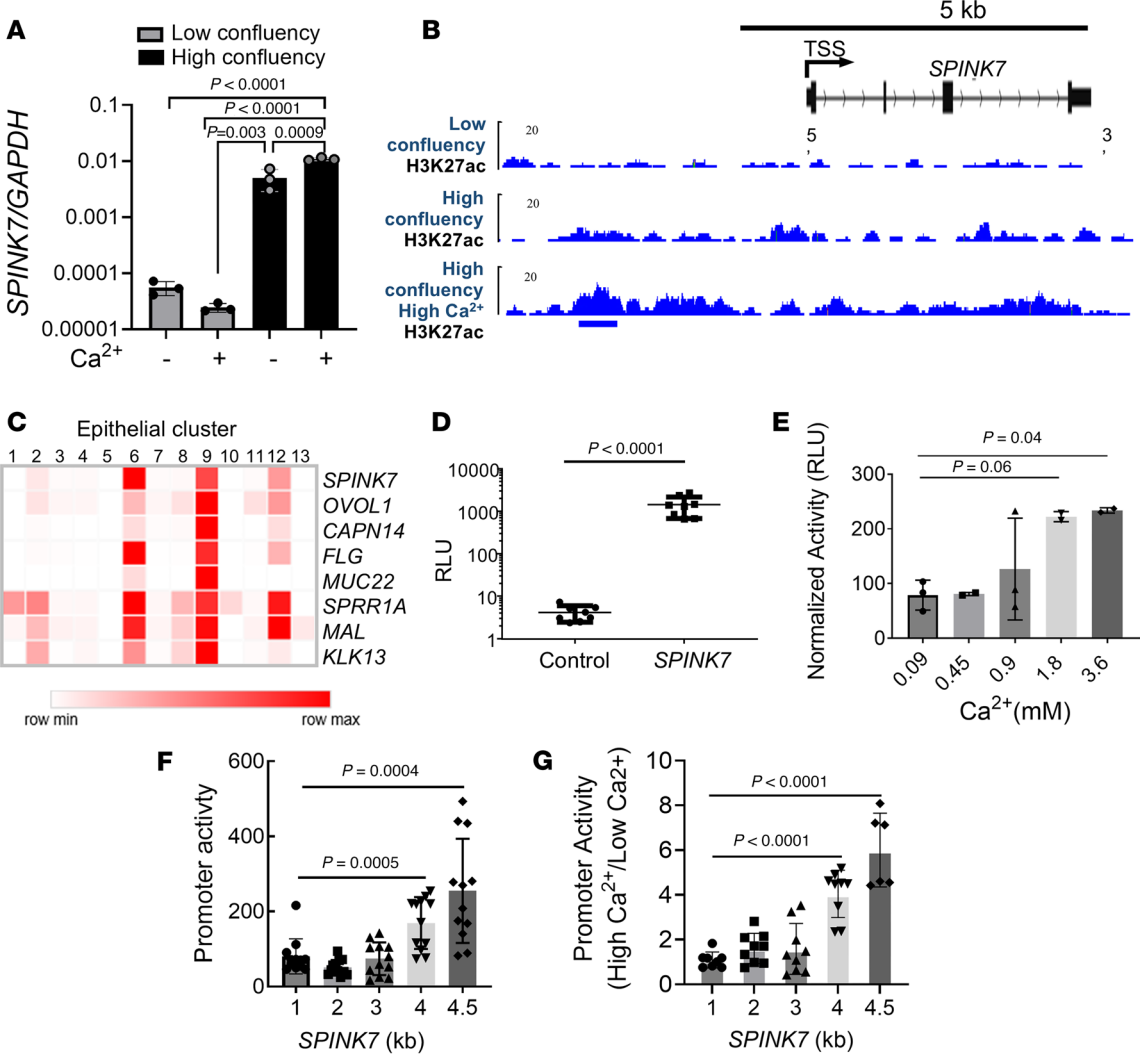
*SPINK7* expression as a function of calcium and cell confluency. (**A**) Quantitative polymerase chain reaction (qPCR) of *SPINK7* expression in EPC2 in cells. (**B**) CHIP peaks H3K27Ac in the promoter region of *SPINK7* in EPC2 cells in the indicated conditions. (**C**) Heatmap depicting the relative expression of the indicated genes in epithelial clusters on the basis of single-cell RNA-seq data of dispersed cells from esophageal control biopsies. (**D**) Promoter activity in cells grown in high calcium and high confluency, cotransfected with either nano-luciferase (nLUC) vector containing the *SPINK7* promoter (*SPINK7*) or a promoterless nLUC vector and with firefly vector to control for transfection efficiency (Control), presented as relative luminescence units (RLU). (**E**) Promoter activity in cells cotransfected with SPINK7-nLUC that were grown in the indicated concentrations of CaCl_2_ and normalized to cells cotransfected with nLUC and firefly vector. (**F**) Promoter activity in cells that were grown in 1.8 mM of CaCl_2_ and cotransfected with nLUC constructs that contain either 0, 1, 2, 3, 4, or 4.5 kb of the *SPINK7* promoter sequence and firefly vector. (**G**) Promoter activity in cells cotransfected with nLUC constructs, containing either 0, 1, 2, 3, 4, or 4.5 kb of the *SPINK7* promoter sequence and firefly vector, and grown in either 0.09 or 1.8 mM of CaCl_2_. The values of the 1.8 mM of CaCl_2_ lysates were divided to the values of the cells cultured in 0.09 mM of CaCl_2_. P values were calculated by 1-way ANOVA.

We analyzed *SPINK7* expression in cultures of esophageal epithelial progenitor cells (EPC2 cells) under conditions that promote cellular differentiation, namely high confluency and high calcium. *SPINK7* expression was more strongly induced under high-confluency conditions compared with low-confluency conditions (Figure 1A). *SPINK7* expression was further increased under high-calcium (1.8 mM) conditions compared with low-calcium conditions in high confluency conditions (0.09 mM; Figure 1A). Epigenetic analysis of the *SPINK7* promoter region of EPC2 cells revealed an increase in H3K27 acetylation marks in the region surrounding *SPINK7* transcription start site (TSS) in high-confluency conditions compared with low-confluency conditions (Figure 1B). The highest levels for the K27 acetylation mark were observed in cells that were grown in high confluency and high calcium (1.8 mM; Figure 1B). These findings indicate that SPINK7 is expressed during esophageal epithelial cell differentiation; we indeed observe this spatial expression of *SPINK7* in differentiated cell clusters based on single-cell RNA-seq data from human esophageal biopsies (24). This analysis demonstrated that, out of 13 identified epithelial cells clusters, SPINK7 is mostly expressed in 3 epithelial clusters together with differentiation markers including *FLG*, *MUC22*, and *SPRR1A* (Figure 1C).

### Identification of the SPINK7 promoter region.

After identifying the regulatory region in the 4.5 kb 5′ region flanking the *SPINK7* TSS using transcriptional and epigenetic data (Figure 1B), we tested the activity of this putative promoter. EPC2 cells were grown at high-confluency and high-calcium (1.8 mM) conditions to induce cell differentiation. Then, cells transiently transfected with a reporter construct containing the presumed promoter region (the 4.5 kb 5′ region flanking the *SPINK7* TSS, referred to henceforth as *SPINK7*) or a control vector. The *SPINK7*-transfected cells had a mean approximately 340-fold increase in luminescence signal compared with cells transfected with the control vector (*P <* 0.0001; Figure 1D). Calcium ions increased promoter activity in a dose-dependent manner and reached a plateau at 1.8 mM of calcium (Figure 1E). No difference was observed in the control vector activity in different calcium concentrations (Supplemental Figure 1A; supplemental material available online with this article; https://doi.org/10.1172/jci.insight.204687DS1). These collective data indicate that this regulatory region of the *SPINK7* gene has calcium-dependent promoter activity.

We subsequently determined the minimal sequence required for promoter activity. We generated reporter constructs containing the presumed promoter region (with either 4.5, 4, 3, 2, and 1 kb 5′ region flanking the *SPINK7* TSS). The promoter activity of all reporter constructs was sufficient to drive at least some activity (Figure 1F) compared with the control construct. The luciferase activity of the cells transfected with the 4.5 kb and 4 kb constructs increased by 3-fold and 2-fold, respectively, compared with the luciferase activity of the cells that were transfected with the 1 kb construct (*P =* 0.0004 and *P =* 0.0001, respectively; Figure 1F). The luciferase activity of the cells transfected with the 3 kb and 2 kb constructs was not different than the luciferase activity of the cells transfected with the 1 kb construct (Figure 1F). In low-calcium conditions, low-promoter activity was observed in all the constructs, with no difference between the 1 kb and the 4.5 kb or 4 kb constructs (Supplemental Figure 1B). For the 4.5 kb and 4 kb constructs, luciferase activities were increased by 7-fold and 4-fold, respectively, in the high-calcium compared with the low-calcium media (*P =* 0.05 and *P =* 0.0003, respectively; Figure 1G). The 3 kb, 2 kb, and 1 kb constructs were not affected by high calcium (Figure 1G). These collective data suggest that induction of cellular differentiation (i.e., high-calcium and high-confluency conditions) promotes *SPINK7* promoter activity via the 4.5 kb region upstream of the *SPINK7* TSS and, more specifically, through the 3–4.5 kb.

### The TF OVOL1 binds to SPINK7 promoter region.

**Figure 2:**
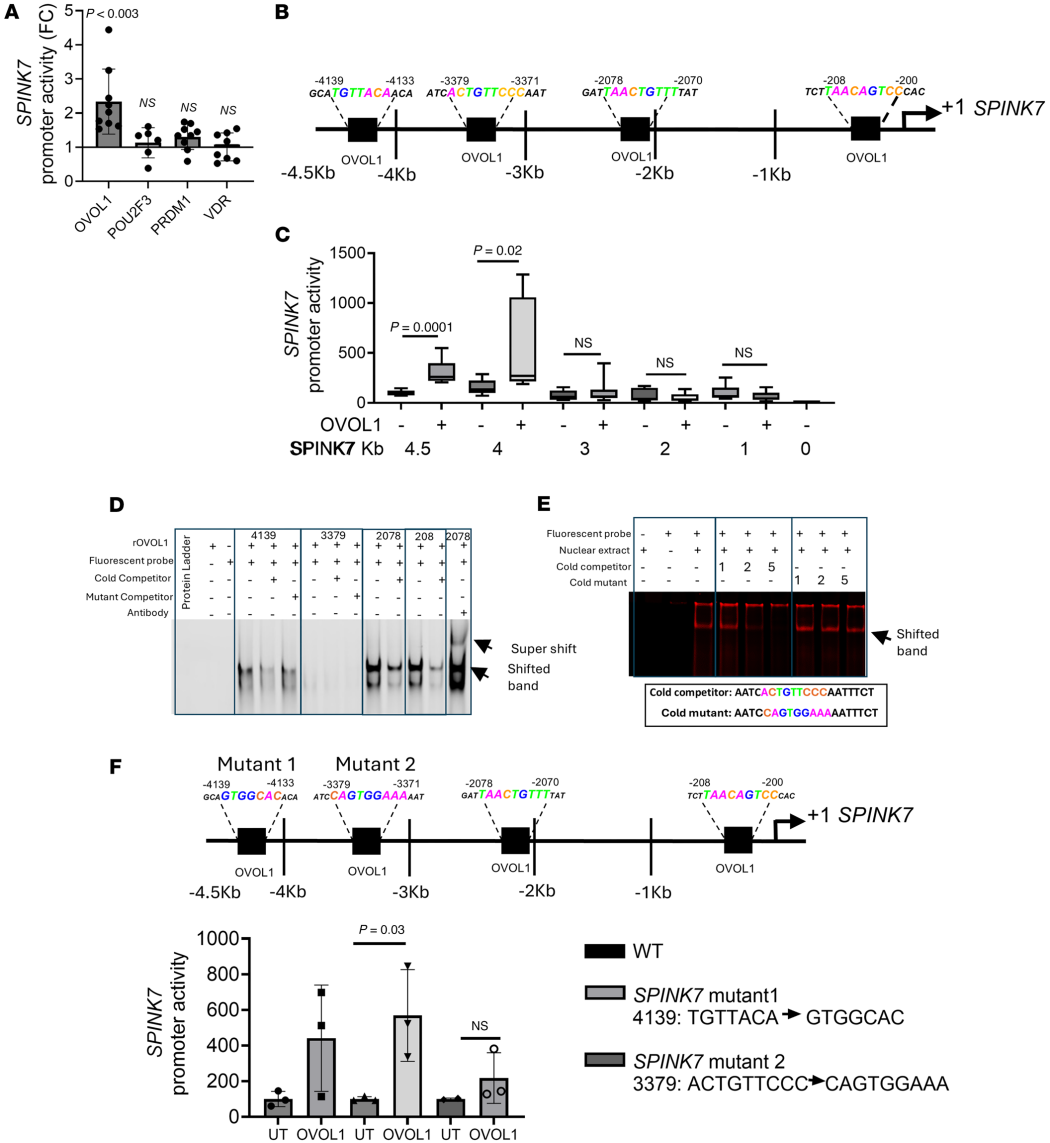
OVOL1 binding to the SPINK7 promoter region. (**A**) Nano-luciferase activity in lysates from cells transfected with *SPINK7* promoter, firefly plasmid, and plasmids encoding for the indicated transcription factors normalized to control lysates from cells transfected with *SPINK7* promoter, firefly plasmid, and an empty plasmid. (**B**) Analysis of the proximal human *SPINK7* promoter sequence identified 4 potential OVOL1 binding sites. Transcription Start Site +1. (**C**) Nano-luciferase activity in lysates cotransfected with either OVOL1 or a control plasmid and with *SPINK7* promoter deletion constructs. (**D**) Representative results from EMSA experiments using recombinant human OVOL1 protein. (**E**) Representative results from EMSA experiment using nuclear extracts from HEK-293T cells transfected with OVOL1 plasmid or a control plasmid. Cold competitors were added in concentrations of 1×, 2×, and 5× compared with the fluorescent probe (presented as 1, 2, and 5). (**F**) Nano-luciferase activity in lysates cotransfected with OVOL1 or a control plasmid (UT) and with the *SPINK7* promoter with either mutated OVOL1 binding site 1 or mutated OVOL1 binding site 2 or WT *SPINK7* promoter. Cells were either left untreated or treated with FICZ (1 μM). *P* values were calculated by 1-way ANOVA.

We next asked which TFs regulate *SPINK7* promoter activity. Analysis of the *SPINK7* promoter using TF binding motifs obtained from the CisBP database (build 1.02) (25) revealed several TFs that are predicted to bind the *SPINK7* promoter. We narrowed this list of TFs to 36 by intersecting with genes that are induced during esophageal epithelial differentiation (as previously reported by us; ref. 11) or with esophageal-specific genes or with genes that are dysregulated in patients with EoE compared with control patients (Supplemental Table 1). We transiently overexpressed 4 resulting candidate TFs that may regulate *SPINK7* expression (i.e., OVOL1, VDR, POU2F3, and PRDM1). Overexpression of VDR (in the presence or absence of the VDR ligand calcipotriol), POU2F3, and PRDM1 did not increase *SPINK7* promoter activity (Figure 2A). In contrast, overexpression of OVOL1 increased *SPINK7* promoter activity by 2.4-fold (*P =* 0.0006; Figure 2A). As a control, Western blot analysis confirmed that OVOL1 protein was overexpressed in the *OVOL1*-transfected cells compared with control cells (Supplemental Figure 2A).

### OVOL1 binds to the SPINK7 promoter and activates SPINK7 expression.

We then examined whether *SPINK7* is a direct OVOL1 target gene in esophageal epithelial cells. We predicted 4 OVOL1 binding sites upstream of the *SPINK7* TSS (Figure 2B). We tested the requirement of this response by examining *SPINK7* promoter activity in the presence or absence of *OVOL1* overexpression (Figure 2B). Overexpression of *OVOL1* induced promoter activity in cells that were transfected with the 4 kb and 4.5 kb constructs, with similar promoter activity (Figure 2C). In contrast, the promoter activity of cells that were transfected with the 3 kb, 2 kb, or 1 kb *SPINK7* constructs were not affected by *OVOL1* overexpression (Figure 2C).

We analyzed the binding of recombinant human OVOL1 protein to a fluorescent DNA probe corresponding to the –4139, –3379, –2078, and –208 regions of the *SPINK7* promoter by Electrophoretic Mobility-Shift Assay (EMSA). OVOL1 shifted the mobility of the fluorescent probe in all probes, except the –3379 probe (Figure 2D). Unlabeled (cold) WT competitors that contain the predicted binding site at –4139, –2078, and –208 bp inhibited the mobility shift (Figure 2D). A mutant cold competitor that contains the predicted binding site at –4139 bp (GTGGCAC) failed to inhibit the mobility shift (Figure 2D). In addition, the administration of a rabbit anti–human OVOL1 antibody resulted in a supershift of the –2078 probe. To examine the possibility that additional cellular cofactors are required for the binding of OVOL1, we generated nuclear extracts of HEK-293T cells overexpressing OVOL1. The nuclear extracts shifted the –3379 probe, whereas the cold WT probe inhibited the shifted band in a dose-dependent manner (Figure 2E). A mutant cold competitor that contains the predicted binding site at –3379 bp (CAGTGGAAA) failed to inhibit the mobility shift (Figure 2E).

We tested the specificity of this response in 2 predicted OVOL1 binding sites that affected the *SPINK7*-nLUC activity when absent from the promoter region (Figure 2C). We constructed mutants in these predicted OVOL1 binding sites and assessed *SPINK7*-nLUC promoter reporter construct activation (Figure 2F). *SPINK7*-nLUC activation was decreased for the OVOL1 binding site mutant at –3379 bp when the ACTGTTCCC sequence was replaced with CAGTGGAAA (Figure 2F). In contrast, there was no effect on SPINK7-nLUC activation for the OVOL1 binding site mutant at –4139 bp when the TGTTACA sequence was replaced with GTGGCAC (Figure 2F). These collective data suggest that OVOL1 binds to all 4 sites in the *SPINK7* promoter and that the –3379 bp region of the *SPINK7* TSS is important for OVOL1-dependent gene expression. Additionally, our data suggest possible involvement of other cofactors regulating the OVOL1-mediated *SPINK7* expression.

### Loss of OVOL1 promotes impaired barrier function and TSLP production.

**Figure 3:**
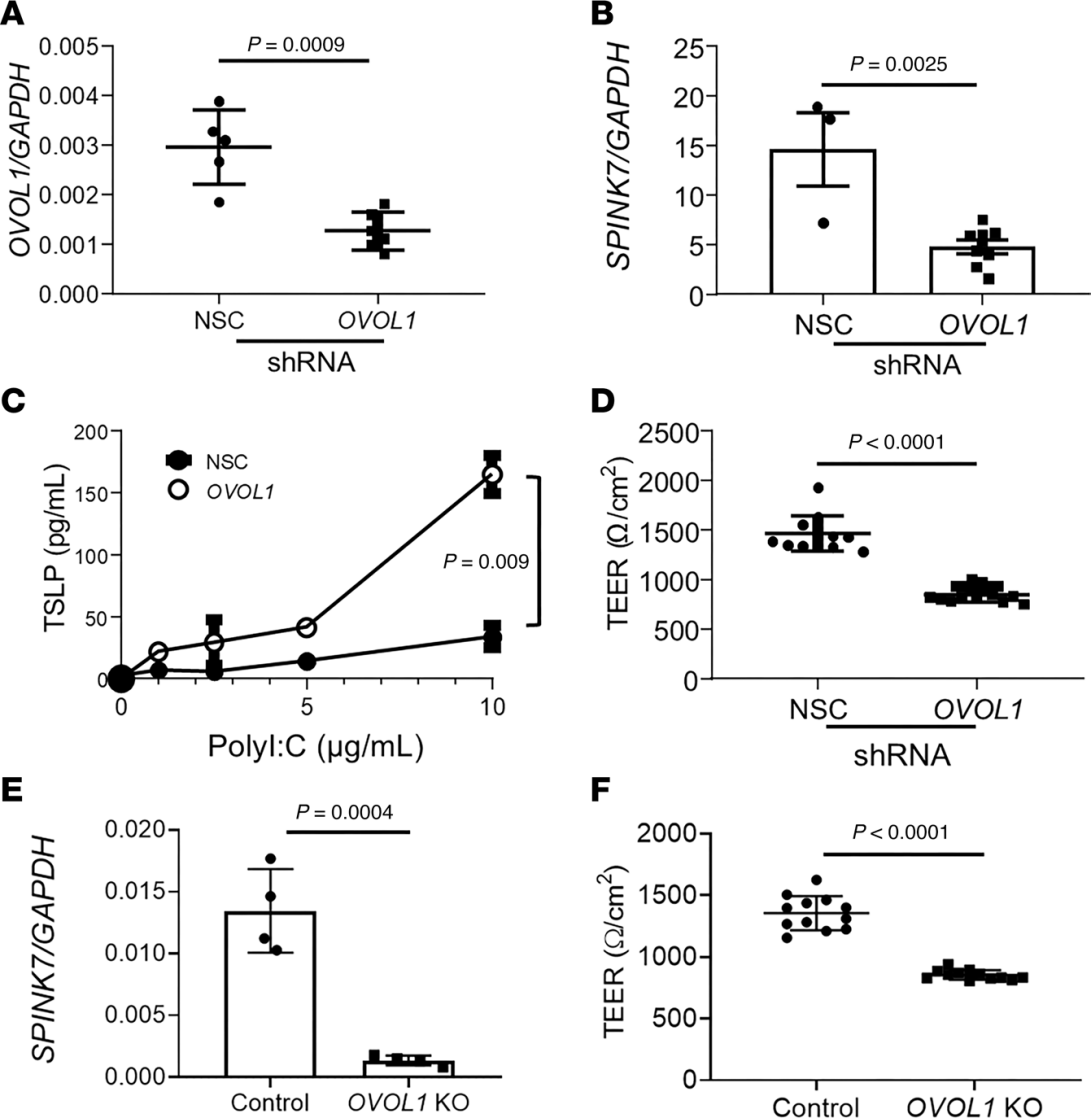
Effect of Loss of OVOL1 on barrier function and TSLP production. (**A**) qPCR analysis of *OVOL1* expression from NSC- treated and *OVOL1*-silenced EPC2 cells. (**B**) qPCR analysis of *SPINK7* expression from NSC-treated and *OVOL1*-silenced EPC2 cells at day 14 of ALI differentiation. (**C**) TSLP release from NSC- treated and *OVOL1*-silenced EPC2 cells that were grown in high-calcium media for 64 hours and then stimulated for 8 hours with the indicated concentrations of polyinosinic-polycytidylic acid (Poly(I:C)). Cell supernatants were assessed for TSLP levels from 3 independent experiments. Data are the mean ± SD. (**D**) TEER (Ω/cm^2^) measurement from NSC-treated, *OVOL1*-silenced EPC2 cells at day 7 of ALI differentiation. Data are the mean ± SD from 3 independent experiments performed in triplicate. (**E**) qPCR analysis of *SPINK7* expression from CRISPR/Cas9 *OVOL1*-KO and control EPC2 cells at day 14 of ALI differentiation. (**F**) TEER (Ω/cm^2^) measurement from *OVOL1* KO and control EPC2 cells at day 7 of ALI differentiation. Data are the mean ± SD from 3 independent experiments performed in triplicate. All *P* values were calculated by Student’s *t* test (unpaired, 2-tailed).

We examined the consequences of depleting *OVOL1* expression in EPC2 cells by stable transduction with a vector expressing either shRNA targeting *OVOL1* or nonsilencing control (NSC) shRNA. *OVOL1-*silenced cells had more than 2-fold reduction in OVOL1 mRNA levels compared with NSC control (Figure 3A and Supplemental Figure 2B). *OVOL1-*silenced cells that were differentiated in air-liquid interface (ALI) culture system had a 3-fold decrease in *SPINK7* expression compared with that of differentiated NSC-treated cells (Figure 3B). *OVOL1-*silenced cells had increased TSLP release compared with NSC-treated cells after Poly(I:C) stimulation (Figure 3C). This is consistent with the phenotype of *SPINK7*-depleted cells, which were previously demonstrated to produce increased TSLP upon Poly(I:C) stimulation (11). *OVOL1* silenced cells displayed barrier impairment as assessed by decreased trans epithelial electrical resistance (TEER; Figure 3D). These data suggest that *OVOL1* expression is critical for maintaining *SPINK7* expression, barrier integrity, and controlling TSLP production by epithelial cells.

We subsequently generated *OVOL1*-KO cells using CRISPR/Cas9 genomic editing (Supplemental Figure 3, A and B). OVOL1 protein expression was undetected in *OVOL1*-KO cells compared with control cells (Supplemental Figure 3C). *OVOL1*-KO cells that were differentiated in ALI culture had a 10-fold decrease in *SPINK7* expression compared with that of differentiated control EPC2 cells (*P =* 0.0004; Figure 3E) and had barrier impairment as assessed by decreased TEER (Figure 3F).

### OVOL1 protein expression is lost in EoE.

**Figure 4:**
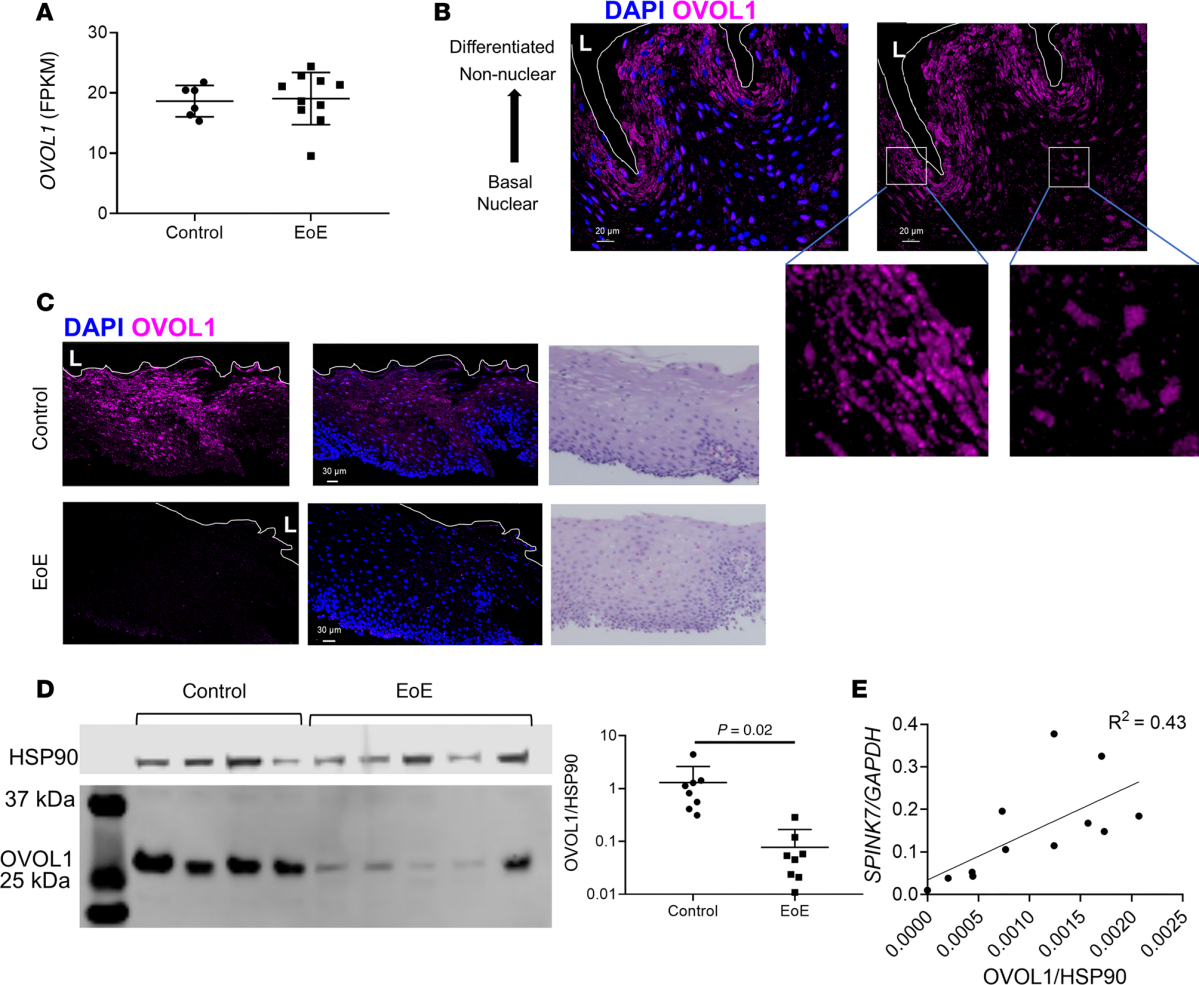
Expression of OVOL1 in EoE biopsies. (**A**) Expression of OVOL1 mRNA in EoE biopsies compared with control biopsies. (**B**) Representative image of immunofluorescence staining of OVOL1 (pink) and DAPI (blue) staining in control biopsy. White line separates the lumen from the epithelium; the lumen side is marked by the letter “L.” Scale bar: 20 µm. (**C**) Representative images of H&E and immunofluorescence staining of OVOL1 (pink) and DAPI (blue) staining in control and EoE biopsies. White line separates the lumen from the epithelium, and the lumen side is marked by the letter “L.” Scale bar: 30 µm. (**D**) Western blot analysis of OVOL1 expression in control and EoE biopsies. The graph on the right shows the OVOL1 expression relative to HSP90. (**E**) Linear regression of OVOL1 protein expression and *SPINK7* mRNA expression from 12 esophageal biopsies (6 patients with EoE and 6 control patients). *R*^2^ and *P* were calculated via sample correlation coefficient and F-test, using GraphPad prism.

Analysis of *OVOL1* mRNA expression did not reveal any difference between EoE biopsies and controls (Figure 4A and Supplemental Table 1). However, when analyzing OVOL1 protein expression in esophageal biopsies, we noted that OVOL1 intracellular localization changed by epithelial compartment. In the differentiating cells closer to the basal membrane, OVOL1 localization was nuclear; whereas, in the differentiated epithelium (the layers of epithelium near the lumen), OVOL1 localization was nonnuclear (Figure 4B). Analysis of OVOL1 protein expression revealed a decrease in protein expression in EoE compared with control biopsies (Figure 4C). Western blot analysis of OVOL1 showed that OVOL1 protein expression was decreased by 10-fold in esophageal biopsies from patients with EoE compared with control patients (Figure 4D). Notably, a higher molecular band of OVOL1 (50 kDa) remained evident in control and EoE biopsies (Supplemental Figure 4A). These collective data suggest that the OVOL1 protein is deficient in the esophagus of patients with EoE compared with control individuals.

### OVOL1 correlates with SPINK7 expression in esophageal biopsies.

Having demonstrated that OVOL1 protein is lost in EoE and that OVOL1 directly binds to SPINK7 promoter, we hypothesized that the level of OVOL1 protein in esophageal biopsies would be correlated with *SPINK7* mRNA expression. From 12 biopsies (*n* = 6 EoE; *n* = 6 controls), we isolated the RNA fraction and the protein fraction and measured the mRNA expression of *SPINK7* and the protein expression of OVOL1 (Figure 4E). This analysis demonstrated a positive correlation (*P =* 0.019) between *SPINK7* mRNA and OVOL1 protein expression (Figure 4E and Supplemental Figure 4B).

### IL-13 and IL-4 inhibit OVOL1 activation.

**Figure 5:**
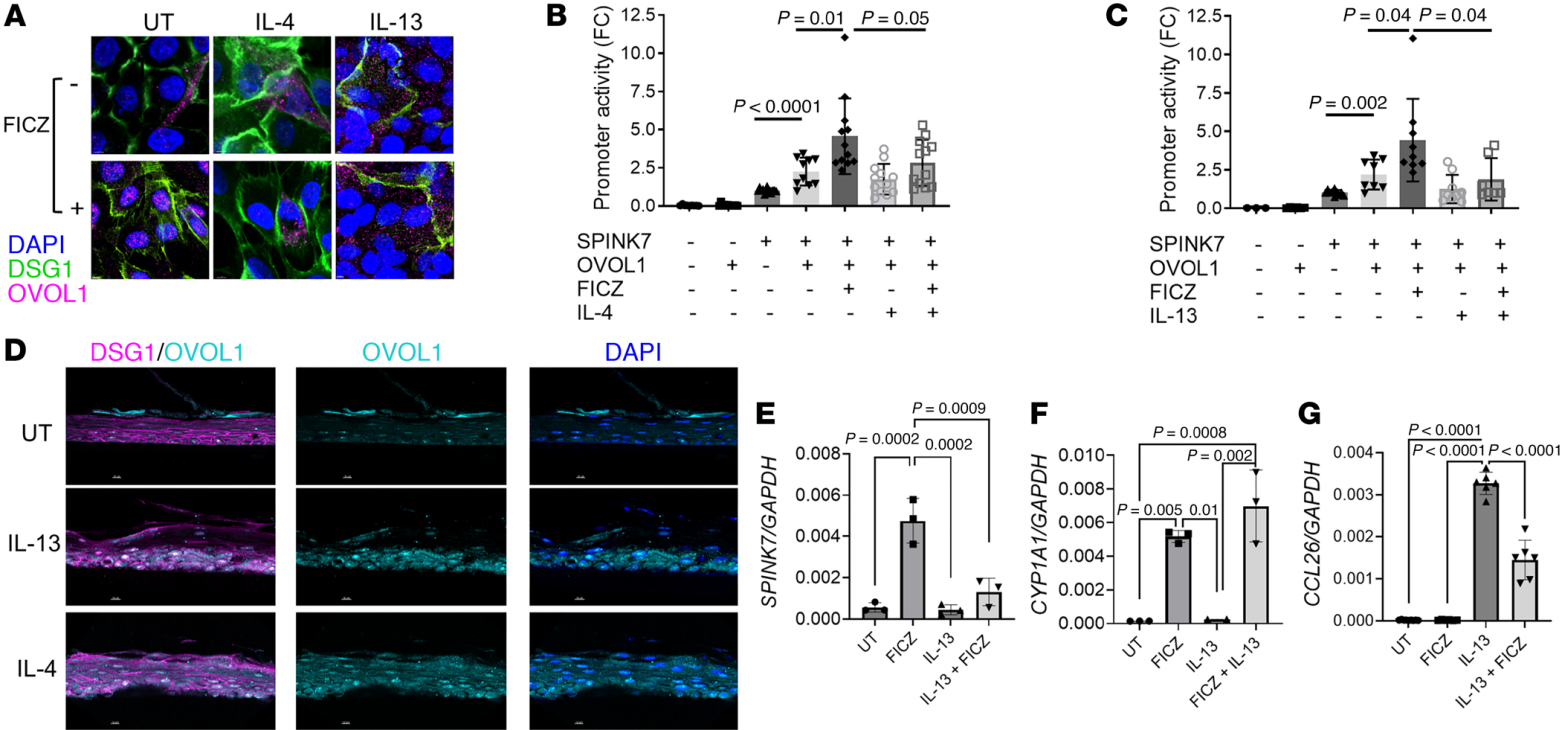
Effect of IL-13 and IL-4 on OVOL1-dependent *SPINK7* expression. (**A**) Representative images of coimmunofluorescence of desmoglein1 (DSG1, green), OVOL1 (pink), and DAPI (blue) stain in OVOL1-overexpressing EPC2 cells that were either left untreated (UT) or treated over night with IL-4 or IL-13 (100 ng/mL) with or without FICZ (1 μm). Scale bar: 5 μm. Promoter activity in lysates triple-transfected with firefly vector and either *SPINK7*-nLUC or nLUC and either OVOL1 or a control plasmid. (**B** and **C**) Cells were either left untreated or treated with 1 μm FICZ, with or without IL-4 (**B**) or IL-13 (**C**). (**D**) Representative images of coimmunofluorescence of DSG1 (pink), OVOL1 (cyan), and DAPI (blue) stain in cells that were differentiated in the ALI model. Cells were either left untreated or treated with IL-4 or IL-13 (100 ng/mL), or IL-4 (100 ng/mL) with FICZ (1 μm). Scale bar: 10 μm. (**E**–**G**) mRNA expression of *SPINK7* (**E**), *CYP1A1* (**F**), or *CCL26* (**G**) normalized to *GADPH* in a monolayer of EPC2 cells that were either left untreated or stimulated with IL-13 (100 ng/mL) and/or FICZ (1 μm). P values were calculated by one-way ANOVA test.

We aimed to determine the effect of IL-4 and IL-13, Th2 cytokines with established roles in atopic diseases, including EoE (8, 26, 27), on OVOL1. First, we investigated the subcellular localization of OVOL1. In unstimulated cells that overexpress OVOL1, OVOL1 was primarily localized to cytoplasmic organelles that bordered the membranal protein desmoglein 1 (DSG1) (Figure 5A). It has been demonstrated that the aryl hydrocarbon receptor (AHR) ligand, 6-Formylindolo[3,2-b]carbazole [FICZ], promotes the translocation of OVOL1 to the cells’ nuclei in keratinocytes (28). FICZ stimulation induced nuclear mobilization of OVOL1, whereas IL-4 or IL-13 stimulations prevented the FICZ-induced OVOL1 mobilization to the nucleus in OVOL1-overexpressing cells (Figure 5A). Consistently with OVOL1 nuclear localization, FICZ induced the promoter activity of *SPINK7*, while IL-4 and IL-13 decreased *SPINK7* promoter activity in OVOL1-overexpressing cells (Figure 5, B and C). Because cells that were differentiated in ALI culture express high endogenous levels of OVOL1, we analyzed the effect of IL-4 and IL-13 on differentiated cells. In unstimulated differentiated cells, OVOL1 was mostly nuclear (Figure 5D). IL-4 or IL-13 stimulation promoted the exit of OVOL1 from the nucleus and decreased OVOL1 expression (Figure 5D and Supplemental Video 1–3). Next, we analyzed the effect of IL-13 on *SPINK7* endogenous expression. FICZ stimulation increased endogenous *SPINK7* expression (Figure 5E), and IL-13 decreased *SPINK7* expression when the cells were stimulated with FICZ (Figure 5E). As a control for FICZ and IL-13 stimulation, their 2 target genes *CYP1A1* and *CCL26* expression were also examined and show the expected increases when used alone (Figure 5, F and G). Interestingly, FICZ stimulation was able to decrease the IL-13–dependent CCL26 expression by 2.7-fold (*P <* 0.0001; Figure 5G).

### IL-13–induced CAPN14 expression depletes OVOL1 protein expression.

**Figure 6:**
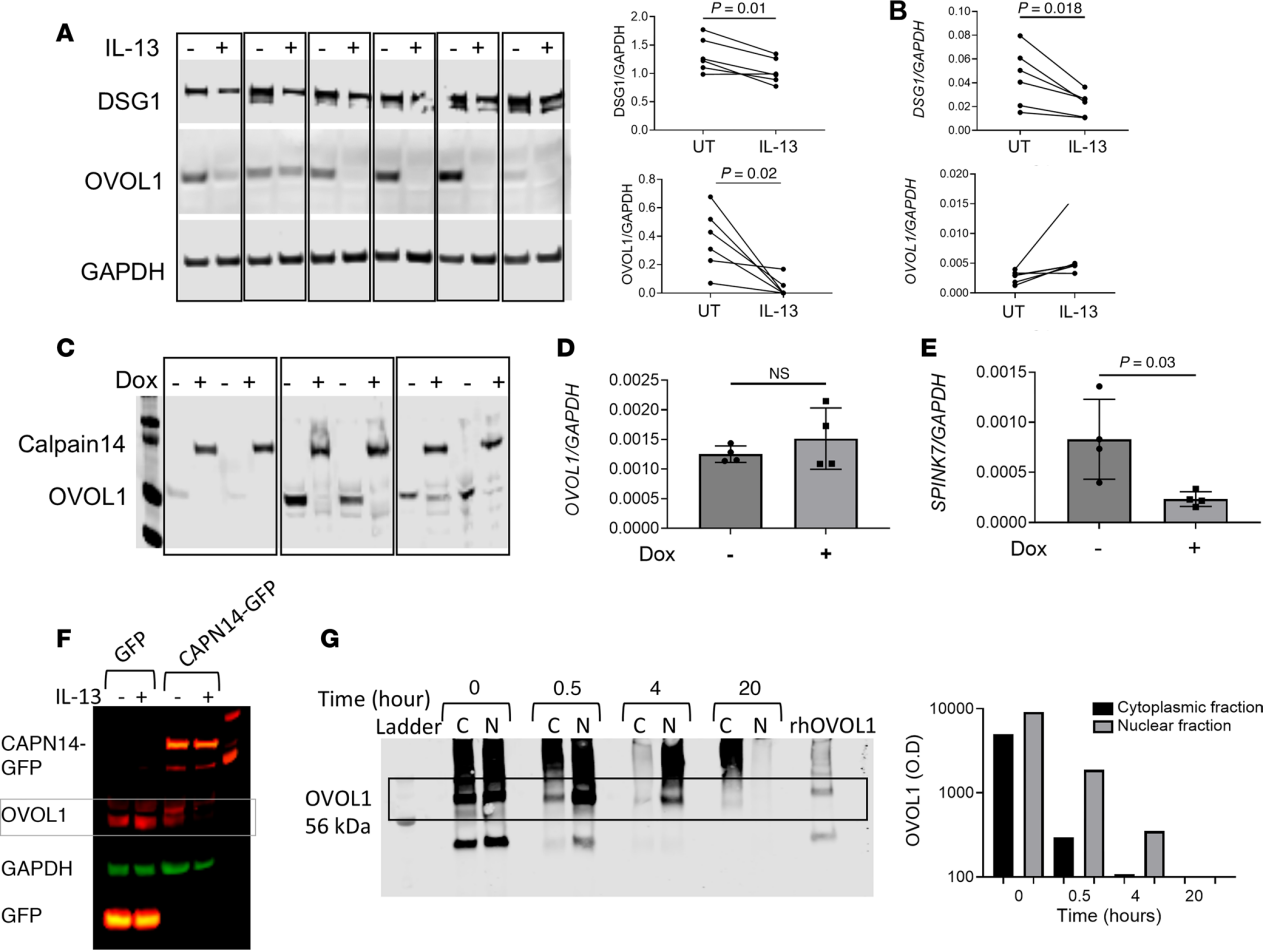
Posttranslational modification of OVOL1 induced by IL-13. (**A**) Western blot analysis of OVOL1, DSG1, and GAPDH expression in differentiated EPC2 cells that were either left untreated (UT) or stimulated with IL-13 (100 ng/mL) for 48 hours. The graphs on the right show quantification of OVOL1 and DSG1 relative to GAPDH with or without IL-13 treatment from paired UT/IL-13 samples from 6 independent experiments. *P* values were calculated by paired Student’s *t* test. (**B**) qPCR analysis of *OVOL1* and *DSG1* mRNA expression in differentiated EPC2 cells that were either left untreated or stimulated with IL-13 (100 ng/mL) for 48 hours; expression is normalized to *GAPDH*. *P* values were calculated by paired Student’s *t* test. (**C**) Western blot analysis of OVOL1 and calpain 14 expression in differentiated EPC2 cells with inducible expression of *CAPN14* expression. *CAPN14* is fused to a flag tag and is induced by doxycycline (Dox) treatment. (**D** and **E**) qPCR analysis of *OVOL1* (**D**) and *SPINK7* (**E**) in differentiated EPC2 with inducible expression of *CAPN14* expression of *CAPN14*; expression is normalized to *GAPDH*. *P* values were calculated by unpaired Student’s *t* test. (**F**) Western blot analysis of OVOL1 in GFP-overexpressing or CAPN14-GFP–overexpressing cells with or without IL-13 treatment. GAPDH was used as a loading control. Anti-GFP was used for detection of GFP and CAPN14-GFP. (**G**) Western blot analysis of recombinant human OVOL1-GST (60 ng) that was either left untreated or incubated with nonnuclear protein fractions (C) or nuclear protein fractions (N) for the indicated times. The graph on the right is a quantification of OVOL1 band intensity (OD) shown from the left. UT, untreated.

Because IL-13 is a major driver of epithelial transcriptional changes, we asked if IL-13 regulates OVOL1 expression. IL-13 stimulation in EPC2 cells differentiated by ALI culture decreased OVOL1 protein expression by an average of 10-fold (*P =* 0.02; Figure 6A). Notably, the decreased OVOL1 expression in the ALI culture system contrasts with the cytoplasmic localization observed in a monolayer of EPC2 following overexpression of OVOL1 and IL-13 stimulation (Figure 5A). We suggest that the differential OVOL1 phenotypes between these 2 model systems is likely to be due to the difference between analyzing the endogenous OVOL1 (Figure 6A) and analyzing exogenous OVOL1 that is under constitutive promoter regulation and is constantly generated by the cells (Figure 6A). As a positive control for IL-13 stimulation, DSG1 protein expression decreased by approximately 20% in IL-13 stimulated ALI cultures compared with untreated ALI cultures (*P =* 0.01; Figure 6A). In contrast, *OVOL1* mRNA expression was unchanged between IL-13–stimulated and control cells (Figure 6B). Consistent with the protein level, *DSG1* mRNA decreased in IL-13–stimulated compared with untreated cells (*P =* 0.018; Figure 6B). These data demonstrate that IL-13 inhibited the nuclear retention of OVOL1 and loss of OVOL1 outside of the nucleus, whereas a nuclear shift of OVOL1 may enable protein stability.

The EoE transcriptome is enriched for proteases and has an imbalance between proteases and protease inhibitors, favoring a proteolytic state (11, 29, 30). Single-cell RNA-seq analysis that was previously performed by us (31) revealed that *CAPN14* and *OVOL1* are coexpressed in the same epithelial cluster in the esophagus (Figure 1C). The epithelial clusters that coexpressed *OVOL1* and *CAPN14* were enriched for *SPINK7* and corresponded to cells that express differentiation markers (i.e., *FLG*, *MUC22*) and esophageal enriched genes (i.e., *MUC22*, *MAL*, *KLK13*; Figure 1C). The expressions of *CAPN14* and *OVOL1* were highly correlated across cells (*r* = 0.92; Spearman correlation). These findings prompted us to hypothesize that calpain-14 may be involved in OVOL1 posttranscriptional regulation. Notably, *CAPN14* is an esophagus-specific protease, encoded by the *CAPN14* gene, which is located in a strong EoE-associated risk locus (i.e., 2p23) (15, 32). *CAPN14* is transcriptionally induced by IL-13 and has been shown to regulate epithelial barrier homeostasis and repair (10, 15). Inducible *CAPN14* expression in differentiated esophageal epithelial cells revealed a marked reduction in OVOL1 protein (Figure 6C). In contrast to OVOL1 protein, *OVOL1* mRNA expression was not affected by inducible *CAPN14* expression (Figure 6D). Induction of *CAPN14* expression decreased *SPINK7* expression by 3.5-fold (*P =* 0.03; Figure 6E). In addition, constitutive expression of *CAPN14*-GFP decreased the expression of OVOL1 compared with that of control GFP vector transduction (Figure 6F), indicating that the reduction in OVOL1 protein expression resulted from *CAPN14* expression and not as a result of doxycycline treatment. Consistently, IL-13 treatment further decreased OVOL1 expression in the *CAPN14*-GFP–overexpressing cells (Figure 6F). We then incubated recombinant OVOL1 protein with either nonnuclear or nuclear extracts; OVOL1 protein degraded more quickly when incubated with nonnuclear proteins compared with nuclear proteins (Figure 6G). These results indicate that OVOL1 is likely degraded outside of the nucleus and remains relatively stable in the nucleus. We therefore suggest that nuclear mobilization of OVOL1 protects OVOL1, whereas IL-13–mediated retention of OVOL1 outside of the cell nuclei, which consequently promotes degradation of OVOL1 by intracellular proteases, such as calpain-14. Accordingly, we incubated recombinant OVOL1 protein with cytosolic or nuclear extracts; OVOL1 protein degraded more quickly with incubation with nonnuclear proteins compared with nuclear proteins (Figure 6G).

## Discussion

Herein, we have interrogated components of the innate immune system of the esophageal epithelium with an initial focus on the mechanisms that regulate a key checkpoint proinflammatory inhibitor SPINK7. We identified elevated histone 3 acetylation marks during cellular differentiation at the *SPINK7* promoter region. We identified binding sites for the C2H2 zinc finger TF OVOL1 in the SPINK7 promoter region and colocalized them with the histone 3 acetylation marks. We demonstrated that OVOL1 directly binds to the *SPINK7* promoter region and induces its expression. Depletion of *OVOL1* decreased *SPINK7* expression and was sufficient to induce epithelial barrier impairment. Both *OVOL1* and *SPINK7* transcripts were expressed by the same epithelial clusters in esophageal biopsies, with the highest expression in epithelial cells that express differentiation markers. Interestingly, while *SPINK7* expression is markedly decreased in biopsies from patients with EoE, *OVOL1* mRNA expression remained the same. In contrast to the mRNA expression of *OVOL*, OVOL1 protein was lost in biopsies from patients with EoE and OVOL1 protein expression correlated with *SPINK7* transcript level in esophageal biopsies. These data suggest the clinical relevance of OVOL1 as a primary upstream regulator of *SPINK7* expression in esophageal tissue and its likely contribution to esophageal epithelial homeostasis and barrier integrity. Furthermore, we demonstrated that IL-4 and IL-13 repress OVOL1 nuclear localization, which consequently inhibits *SPINK7* expression. Moreover, we showed that OVOL1 protein is more stable in the cell nuclei. Consistent with this finding, IL-13 stimulation decreased OVOL1 protein expression but not *OVOL1* transcript. The cysteine protease calpain-14 (which is induced by IL-13 stimulation, also the product of a major EoE susceptibility locus [2p23]; ref. 10) was found to decrease OVOL1 protein expression. These data suggest the possible role of IL-13 and calpain-14 in posttranscriptional regulation of OVOL1 in context of the disease state.

We provide evidence that the TF OVOL1 is a regulator of esophageal epithelial barrier formation. OVOL1 is an enriched esophageal TF that is induced during esophageal epithelial differentiation (28, 33). OVOL1 regulates epithelial differentiation during hair formation, spermatogenesis, and skin development (34, 35) by inducing the expression of barrier genes such as *FLG* and *LOR* (36–39). OVOL1 also regulates reepithelialization of cancer cells (40, 41). In addition, variants in the *OVOL1* gene are associated with atopic dermatitis, a type 2 allergic disease with strong similarities to EoE, including impaired barrier function (20–23). Our data demonstrate that *OVOL1* depletion decreases *SPINK7* expression and promotes impaired esophageal barrier function and TSLP production.

Interestingly, OVOL1 protein expression was lost in esophageal biopsies from patients with EoE compared with controls. We suggest that OVOL1 has a key role in regulating esophageal homeostasis and suppression of inflammation. While the molecular mechanisms that regulate *SPINK7* expression are mostly obscure, to date, 1 study has reported a potential role for p53 and DNA damage in regulating SPINK7, has been reported (42), calling attention to examining this in type 2 immunity.

Our data suggest that IL-13 and calpain-14 promote the loss of OVOL1 during disease state at least in part. We provide evidence that IL-13 posttranscriptionally regulates OVOL1 by 2 mechanisms; first, it does so by inhibiting OVOL1 nuclear translocation, which induces rapid degradation of OVOL1 and prevents OVOL1 from binding to its target genes (e.g., *SPINK7*) and, second, by inducing calpain-14 expression, which consequently degrades OVOL1 and prevents *SPINK7* expression. Whether OVOL1 is directly cleaved by calpain-14 is uncertain. These data place IL-13 and calpain-14 as upstream regulators of SPINK7, and their overexpression in patients with EoE impairs epithelial differentiation and induces barrier breach. The importance of IL-13 in EoE is highlighted by the recent approval of dupilumab for EoE treatment (27, 43, 44). Dupilumab inhibits the IL-4 and IL-13 signaling pathways by blocking the shared receptor subunit IL-4Rα (45). Thus, inhibiting this signaling pathway may restore downstream *SPINK7* expression and consequently epithelial integrity. Indeed, treatment with the anti–IL-13 drug cendakimab normalized the expression of *SPINK7* in patients with EoE (46). The cysteine protease calpain-14 is amenable to inhibition (47), thus, making this protease an attractive target for pharmacological intervention as well. IL-13 has broad effects on multiple cell types, including epithelial cells, with the OVOL1/SPINK7 axis representing one of its downstream epithelial targets. Overexpression of IL-13 in allergic diseases, including EoE, is driven by upstream alarmins such as IL-33 and TSLP, which initiate and amplify type 2 immune responses (13, 48–50). IL-33 signaling in EoE is modulated by the soluble IL-33 receptor ST2 (sST2), which can act as a decoy receptor to limit IL-33 activity, and IL-13 itself can enhance sST2 production, thereby dampening upstream IL-33–driven responses (51–53). Importantly, we demonstrated that loss of OVOL1, or its downstream target gene, SPINK7 (11), induces overproduction of TSLP, indicating that the consequences of OVOL1/SPINK7 loss provides positive feedback that amplifies and sustains type 2 inflammation despite the presence of counter-regulatory mechanisms. Notably, the mechanism that regulate TSLP overproduction in the setting of loss of OVOL1/SPINK7 is unknown and likely an indirect consequence of impaired epithelial barrier function rather than a direct effect.

Interestingly, when we analyzed the expression of OVOL1 by Western blot analysis in differentiated epithelial cells or in esophageal biopsies, we identified 2 bands that correspond to OVOL1. CRISPR-Cas9 deletion of *OVOL1* resulted in diminishing of all the detectable molecular weights forms of OVOL1, demonstrating that OVOL1 is subjected to transcriptional or posttranscriptional changes. Splicing events in TFs that regulate epidermal differentiation including OVOL1 were identified to be regulated by DDX21 (54). Since both the esophagus and the skin are composed of squamous epithelium, it is tempting to speculate that DDX21 also serves as a regulator of differentiation in the esophageal epithelium via OVOL1 splicing. Miao et al. identified the RNA helicase DDX21 as an upstream regulator of *OVOL1* mRNA splicing in the skin (54). The authors demonstrated that the differentiated epithelium of the skin contains higher glucose concentration, which switches the role of DDX21 from RNA helicase to a regulator of mRNA splicing. Thus, splicing mechanisms may explain the identification of the 2 molecular weight forms of OVOL1 in the esophageal epithelium.

We acknowledge that this study, which includes human cells and clinical samples, is limited by the lack of investigation into the complex interactions between epithelial cells and esophageal resident cells and infiltrating cells in the context of OVOL1 dysfunction and *SPINK7* loss. In this regard, in vivo studies will be essential to capture the consequences of altering the OVOL1/*SPINK7* pathway within the entire esophagus and to further advance our understanding of its role in disease pathogenesis. Additionally, we focused on the role of OVOL1 in regulating *SPINK7* expression, although our analysis identified 36 TFs that may bind the SPINK7 promoter. Thus, additional TFs may regulate SPINK7 and could also contribute to its loss in EoE. Finally, we investigated the role of IL-13 and calpain-14 as upstream regulators of the OVOL1/*SPINK7* pathway, while other upstream factors not examined in this study, such as environmental signals that influence AHR signaling, cellular stress, and additional immune cell-derived mediators (55–61), may also contribute to OVOL1/*SPINK7* regulation.

In conclusion, we found that binding of OVOL1 to the *SPINK7* promoter was required for its activity. Furthermore, we demonstrated that the type 2 cytokines IL-4 and IL-13 repressed OVOL1 activation. Additionally, the product of the chief EoE susceptibility locus (2p23) calpain-14 (15), an intracellular regulatory protease induced by IL-13 in esophageal epithelial cells (10), was involved in posttranscriptional modification of OVOL1 levels. Translational studies identified a marked loss of OVOL1 protein expression in esophageal biopsies of patients with EoE compared with control patients. In contrast to OVOL1 protein, the mRNA transcript of OVOL1 is comparable between EoE biopsies and control biopsies. It has not escaped our attention that our findings have implications for other type 2 allergic diseases such atopic dermatitis, a disease genetically linked to *OVO1* variants, as well as Netherton’s Syndrome, caused by mutations in *SPINK5* (14, 17, 20–22, 62). Given these collective observations, our findings extend the homeostatic antiinflammatory and sensing regulatory mechanism of the esophagus (2), we propose that homeostasis in the esophagus is controlled by OVOL1 transcriptional activity, which is suppressed by IL-13 and calpain-14 during allergic inflammation. A deeper understanding of the reported findings and their in vivo relevance are warranted.

## Methods

Further information can be found in Supplemental Methods.

### Sex as a biological variable.

Sex was considered as a biological variable in the design and interpretation of these studies, but clinical sample availability was constrained by the known male predominance of EoE.

### Identification of the SPINK7 promoter.

The 4.5 kb region was chosen based on bioinformatics analysis of transcriptional and epigenetic data from ENCODE (Encyclopedia of DNA Elements) and BioWardrobe (Cincinnati Children’s Hospital Medical Center [CCHMC] Epigenetic Database). Cross analysis of these databases has shown that the 4.5 kb region consists of highly conserved sites enriched with histone acetylation marks (H3K27ac) and overlapped with DNase clusters in multiple relevant cellular contexts. The BioWardrobe database (internal unpublished data) has shown that a region of 1.8 kb is enriched with H3K27ac marks at 2 kb upstream of the TSS. The 4.5 kb, noncoding putative promoter sequence (without untranslated region) was obtained from the ENCODE UCSC Genome Browser of the Human genome 2013 database (hg38_dna range), and the coordinates are chromosome 5:148307922-148312422.

### Construction of plasmids.

Promoter constructs were created by cloning the immediate 4.5 kb region adjacent to the 5′ TSS of *SPINK7* into the promoterless Nano-luciferase reporter vector pNL1.1-NL (Promega). The 4.5 kb sequence and subsequent constructs were created by using primers with the restriction enzyme sites *KpnI-HF* and *XhoI*. We utilized SnapGene software that employed In-Fusion cloning techniques. Cloning was performed with In-Fusion HD methods (Clonteck, Takara Bio Company). pNL1.1-NL is defined as the empty vector (EV). Postcloning with the sequence of interest is termed as SPINK7 (4.5 kb). The full-length SPINK7 consists of 4.5 kb, and shorter lengths were defined as SPINK7 1–3 kb from TSS. Mutations of OVOL1 binding sites were performed using QuikChange Lightning site-directed mutagenesis kits (Agilent).

### Generation of CRISPR/Cas9-KO EPC2 cells.

Guide RNA (gRNAs) complementary to the *OVOL1* open reading frame sequences and located directly 5′ of a protospacer adjacent motif (PAM) were identified (*OVOL1* gRNA: 5′-TCTCGCCGCGCTCCTCGTCG-3′; ref. 63), and the following oligonucleotides (*OVOL1* 5′-CACCGCTCGCCGCGCTCCTCGTCG-3′ and 5′-AAACCGACGAGGAGCGCGGCGAGC-3′) were annealed and ligated into the *BbsI* restriction site of plasmid pX459M2 (obtained from CCHMC Transgenic Mouse and Gene Editing Core Facility) to produce pX459M2-OVOL1g3 and pX459M2-CAPN14g3, respectively. EPC2 cells were transfected with pX459M2, pX459M2-OVOL1g3, or pX459M2-CAPN14g3 using Viromer (Origene) according to the manufacturer’s protocol. Transfected cells were selected and cloned, and gDNA was isolated, amplified, and sequenced as previously described (11). OVOL1 protein expression was determined by rabbit anti-human OVOL1 antibody (Sigma-Aldrich and LSbio).

### Nuclear and nonnuclear extraction and Western blotting.

Proteins from cell cultures were extracted with RIPA buffer (Pierce) with protease and phosphatase inhibitors. Loading buffer (Life Technologies) was added, and samples were heated to 95°C for 5 min, subjected to electrophoresis on 12% NuPAGE Bis-Tris gels (Life Technologies), transferred to nitrocellulose membranes (Life Technologies), and visualized using the Odyssey CLx system (LI-COR Biosciences) with IRDye 800RD goat anti-rabbit (LI-COR Biosciences), and IRDye 680RD goat anti-mouse (LI-COR Biosciences) secondary antibodies. The primary antibodies were rabbit anti-OVOL1 (Sigma Aldrich; HPA003984) or rabbit anti-OVOL1 (LifeSpan Biosciences; LS-C435365), mouse anti-HSP90 (Origene; TA500494), mouse anti–desmoglein-1 (Sigma Aldrich; sc-137164), and rabbit anti-LaminB1 (Proteintech 12987-1-AP). Blots were quantified using the Image Studio software (LI-COR Biosciences).

To prepare nuclear and cytoplasmic extracts, cells were harvested in cold hypotonic lysis buffer (20 mM Tris-HCl, pH 7.4; 10 mM NaCl; 3 mM MgCl_2_), and the suspensions were incubated on ice for 15 minutes. Following addition of NP-40 to a final concentration of 0.5%, cell suspensions were homogenized by vortexing for 10 seconds and centrifuged at 4°C for 10 minutes at 14,000 × *g* to pellet nuclei. Cytoplasmic fractions contained in the supernatant were collected, and nuclear pellets were washed twice with PBS prior to resuspension in extraction buffer (10 mM Tris, pH 7.4; 2 mM Na_3_VO_4_, 100 mM NaCl, 1% Triton X-100, 1 mM EDTA, 10% glycerol, 1 mM EGTA, 0.1% SDS, 1 mM NaF, 0.5% deoxycholate, 20 mM Na_4_P_2_O_7_, purchased from Fisher #FNN0011). Nuclear suspensions were incubated on ice for 30 minutes with vortex every 10 minutes and then centrifuged at 4°C for 30 minutes at 14,000 × *g*. Nuclear proteins contained in the supernatant were collected. Nuclear and cytoplasmic extracts were aliquoted and stored at –80°C.

### Electrophoretic mobility shift assays.

Single-stranded oligonucleotides containing the binding sequences of interest were obtained from Integrated DNA Technologies and incubated with either 5′-IRDye 700 labeled or unlabeled complementary strands in annealing buffer (composition) for 5 minutes at 95°C and then allowed to slowly return to room temperature to generate fluorescent and nonfluorescent double-stranded oligonucleotide probes. Fluorescent probe (40 ng) was incubated in binding buffer (Tris, NaCl, poly[dI:dC], NP-40, glycerol) with 250 ng total protein from cell extracts or 120 ng recombinant GST-tagged OVOL1 protein (Abnova #H00005017-P01) and nonfluorescent competitor containing the wild-type or mutant binding site for 30 minutes at room temperature and cross-linked at 120 mJ/cm^2^. Antibody against OVOL1 was added after cross-linking and incubated at room temperature for 15 minutes before loading samples. Binding reactions were resolved on 6% native PAGE gel electrophoresis, and fluorescent probes were detected using a LICOR Odyssey CLx system. Fluorescent IRDye 700–labeled probe sequence 5′-AATCACTGTTCCCAATTTCT-3′; unlabeled (cold) WT competitor sequence: 5′-AATCACTGTTCCCAATTTCT-3′; cold mutant sequence: 5′-AATCCAGTGGAAAAATTTCT-3′; 4139: 5′-CATTTCTGTTACATTAGGAT-3′; 4139 mutant: 5′-CATTTCGTGGCACTTAGGAT-3′; 2078: 5′-GTAGATTAACTGTTTATGTT-3′; and 208: 5′-ATTCTTAACAGTCCCACCTT-3′.

### TSLP levels.

The levels of TSLP were measured in the cellular supernatant by TSLP ELISA (Biolegend).

### Statistics.

Raw luciferase data were measured as relative luminescent units (RLU), defined by the ratio of Nano-luciferase (NL) reporter activity to the Firefly (FF) activity (NL/FF). Normalized data are defined by the ratio of raw data of the promoter activity (NL/FF) to the average of the EV activity (NL/FF). Statistical analysis was completed with GraphPad PRISM. One-way ANOVA and 2-tailed Student’s *t* test were performed.

### Study approval.

Samples were obtained following informed consent under the auspices of the IRB of CCHMC (no. 2008-0090).

### Data availability.

Values for all data points shown in graphs and values behind any reported means are available in the Supporting Data Values file.

## Author contributions

NPA conceptually led the study, supervised the study, designed and performed experiments, analyzed data, and wrote the manuscript. AMK and SSB designed and performed experiments and analyzed data. KR performed and analyzed data, MR, MP, JMC, and MB designed and performed experiments. ATD, DM, CF, and XC assisted in experimental procedures. LCK and MTW assisted in conceptual design of the study. MER conceptually lead the study, supervised the study, and wrote the manuscript.

## Conflict of interest

MER is a consultant for Pulm One, Spoon Guru, ClostraBio, Serpin Pharm, Celldex, Uniquity Bio, EnZen Therapeutics, and Guidepoint, and has an equity interest the first seven plus Santa Ana Bio, and royalties from reslizumab (Teva Pharmaceuticals), PEESSv2 (Mapi Research Trust), and UpToDate. MER and NPA are inventor of patents owned by Cincinnati Children’s Hospital Medical Center (US 10,821,094 B2).

## Funding support

This work is the result of NIH funding, in part, and is subject to the NIH Public Access Policy. Through acceptance of this federal funding, the NIH has been given a right to make the work publicly available in PubMed Central.

- NIH R01 AI045898, U19 AI070235, R01 DK140127.
- The Cincinnati Children’s Hospital Medical Research Foundation.
- Campaign Urging Research for Eosinophilic Disease (CURED).
- Sunshine Charitable Foundation and its supporters, Denise and David Bunning.

## Supplementary Material

Supplemental data

Unedited blot and gel images

Supplemental table 1

Supplemental video 1

Supplemental video 2

Supplemental video 3

Supporting data values

## Version 1 04/28/2026

In-Press Preview

## Version 2 06/22/2026

Electronic publication
