# Supplementary material for: IL-13 and calpain-14 suppress the expression of SPINK7 by regulating OVOL1 in eosinophilic esophagitis
Source: JCI Insight. 2026 Apr 28;11(12):e204687. doi: 10.1172/jci.insight.204687 (PMC13313498; doi:10.1172/jci.insight.204687)
Supplement: Supplemental data [file jciinsight-11-204687-s223.pdf]

Supplementary Information

IL-13 and Calpain-14 Suppress the Expression of SPINK7 by Regulating OVOL1 in Eosinophilic Esophagitis

Nurit P. Azouz[1,2], Andrea M. Klingler[1], Sierra S. Beach[1], Kalen Rossey[1], Mark Rochman[1], Misu Paul[1], Julie M. Caldwell[1], Michael Brusilovsky[1], Alexander T. Dwyer[1], Xiaoting Chen[1,3], Daniel Miller[1,3], Carmy Forney[1,3], Leah C. Kottyan[1,2,3], Matthew T. Weirauch[1,2,3,4] and Marc E. Rothenberg[1,2]

[1]Division of Allergy and Immunology, Cincinnati Children's Hospital Medical Center, Department of Pediatrics, University of Cincinnati College of Medicine, Cincinnati, Ohio, USA
[2]Department of Pediatrics, University of Cincinnati College of Medicine, Cincinnati, OH, USA
[3]Center for Autoimmune Genomics and Etiology, Cincinnati Children's Hospital Medical Center, University of Cincinnati College of Medicine, Cincinnati, Ohio, USA
[4]Divisions of Human Genetics, Biomedical Informatics, and Developmental Biology, Cincinnati Children's Hospital Medical Center, University of Cincinnati College of Medicine, Cincinnati, Ohio, USA

Supplementary Materials and Methods

Transient transfection and luciferase activity assay

Human esophageal epithelial progenitor cells (EPC2) are immortalized cell lines that were donations from the Dr. Anil Rustgi Lab (Columbia University, NI). EPC2 cells were cultured in Keratinocyte Serum-Free Medium (KSFM). The KSFM medium was supplemented with epidermal growth factor (EGF, 1 ng/mL), bovine pituitary extract (BPE 50 mg/mL), and 1X penicillin/streptomycin (Invitrogen). EPC2 cells were grown for 3-4 days until they reached 80-90% confluency. Cells were then harvested by addition of trypsin/EDTA (Invitrogen) and incubated for 3-5 min at 37˚C. Next, soybean trypsin inhibitor (250 mg/L in 1X DPBS) was added, and cells were pelleted at 300 x *g* for 5 min. EPC2 cells were seeded on day 0 at 200,000 per 4-well plate in two conditions: low calcium (KSFM medium alone at $CaCl_2$ 0.09 mM) and high calcium (KSFM at $CaCl_2$ 1.8 mM). Twenty-four hours later (day 1) cells were transiently transfected at >95% density with Opti-MEM (ThermoFisher) and Mirus TransIT-2020 (Mirusbio, Madison, WI) according to the manufacturer instructions. We used 2 µL of TransIT-2020 per 500 ng of construct DNA and 50 ng Firefly DNA (1:20 dilution). Cells were co-transfected with 1000 ng pNL1.1 containing the *SPINK7* promoter sequence or promoterless empty vector (EV) pNL1.1-NL with 50 ng Firefly luciferase pGL3 vector (Promega). Firefly, a PGL3 vector (Promega), was employed as an internal control for transfection. On day 2, cells were incubated at 37°C, and the medium was changed with the appropriate calcium conditions. On day 3 (48 h post-transfection), Nano and Firefly luciferase activities were measured in relative light units (RLU) by luciferase assay NanoDLR kit (Promega) via Synergy 2 and Synergy H1 Multi-Mode Microplate Reader (BioTek Instruments, Winooski, VT). To

control for transfection efficiency, Nano-luciferase activity was normalized to Firefly-luciferase, and then the activity was normalized to control promoterless transfected cells for each sample transfection to account for variance per well. All assays were conducted in triplicate in 3 independent experiments.

Endogenous *SPINK7* expression

To determine endogenous *SPINK7* expression, EPC2 cells were plated at high density (250,000 cells/well in a 48-well plate) in KSFM media with 1.8 mM $CaCl_2$. For plating at low density, 250,000 cells/well were grown in a 6-well plate in KSFM media with 1.8 mM $CaCl_2$. RNA was isolated with Quick-RNA Micro-prep (Zymo; Irvine, CA). ProtoScript First Strand cDNA Synthesis kit (NEB; Ipswich, MA) was employed according to the manufacturer instructions to obtain RT-PCR data. For air-liquid interface (ALI) differentiation, cells were grown as previously described (11, 64). Briefly, 150,000 cells/well were plated in a transwell system with 24-well plate. After 48 h, media was replaced with a high calcium media (1.8 mM $CaCl_2$). On day 8, the media was aspirated from the top chambers; on day 12, the cells were stimulated; and on day 14, the cells were harvested.

mRNA extraction and quantitative RT-PCR

Total RNA was isolated from cells with the RNeasy mini kit (Qiagen) according to the manufacturer's protocol. For RNA sequencing experiments, RNA was treated with On-Column DNase Digestion kit (Qiagen) according to the supplied protocol. cDNA was synthesized with the ProtoScript synthesis kit (New England Biolabs). qPCR was

performed using a 7900HT Fast Real-Time PCR system from Applied Biosystems (Life Technologies) with FastStart Universal SYBR Green Master mix (Roche Diagnostics Corporation) by using the following primer sets: *GAPDH* (forward 5′-TGGAAATCCCATCACCATCT, reverse 5’-GTCTTCTGGGTGGCAGTGAT), *SPINK7* (forward 5′-GCATTTACAAGAAGTATCCAGTGGT, reverse 5’-TCGTGAAGAAACTGAACTCTTCC), *DSG1* (forward 5’-TGGCTACATTTGCAGGACAA, reverse 5’-CGGTTCATCTGCGTCAGTAG), *OVOL1* (forward 5’-CAAAAGAGGTCCCCAGAACA, reverse 5’-AGGAGCCTTCCTCTCAGGTC), *CYP1A1* (forward 5’-GACAGATCCCATCTGCCCTA, reverse 5’-GGTTGATCTGCCACTGGTTT), *CCL26* (forward 5’-CTCCCAGCGGGCTGTGATATTC, reverse 5’-GGGTCCAAGCGTCCTCGGAT).

Analysis of epithelial clusters

Expression data of selected genes according to epithelial cluster was obtained from next-generation single-cell RNA sequencing of human esophageal biopsies deposited under GSE201153 (65). Clustering of epithelial cells was described previously (31).

***OVOL1* gene silencing by shRNA**

Lentiviral shRNA vectors against OVOL1 (MISSION shRNA, Sigma-Aldrich, clone NM_004561, TRCN00000257410, TRCN0000229665, TRCN0000229664, and TRCN0000229666) and a control vector that targets no known mammalian genes (SHC002 SIGMA MISSION® pLKO.1-puro Non-Mammalian shRNA Control) were used. EPC2 cells grown in KSFM media were transduced. Twenty-four hours after transduction,

cells were selected for stable integration using puromycin (1 μg/mL). After ALI differentiation, gene silencing efficiency of target vectors in transduced cells was assessed by quantitative PCR relative to that of cells transduced with non-silencing control (NSC) shRNA.

Immunofluorescence

Cells that were grown in ALI cultures on top of a membrane were fixed with 10% formalin and embeded in paraffin to preserve the architecture. Formalin-fixed, paraffin-embedded (FFPE) samples were sectioned and de-paraffinized using xylene and then subjected to graded ethanol washes. Heat-induced epitope retrieval in sodium citrate buffer (10 mM sodium citrate, 0.05% Tween 20, pH 6.0) was used. Slides were blocked in 1X phosphate-buffered saline (PBS) with 10% goat serum for 1 h followed by a 1-h incubation at room temperature in the following primary antibodies: rabbit anti-human OVOL1 (Sigma Aldrich; HPA003984) and mouse anti–human DSG1 (Santa Cruz Biotechnology; sc-137164). Slides were then washed, incubated for 1 h at room temperature in secondary antibodies (donkey anti–rabbit Alexa Fluor 488, donkey anti-Rabbit Alexa Fluor 568, and goat anti–mouse Alexa Fluor 594 [Thermo Scientific]) and mounted with 4',6-diamidino-2-phenylindole (DAPI) Fluormount-G (Novus Biological). Images were obtained using the NIKON A1RSi confocal microscope.

Supplementary Figures

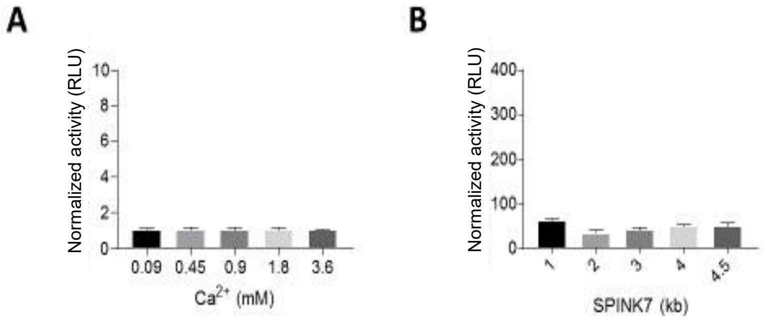

Supplementary Figure 1. Regulation of the *SPINK7* promoter. A. Promoter activity in lysates co-transfected with promoterless nano-luciferase (nLUC) and firefly vector that were grown in the indicated concentrations of $CaCl_2$. Promoter activity was determined by nLUC measurements relative to firefly measurements and normalized according to the promoter less nLUC measurements. Promoter activity is presented as relative luminescence units (RLU). **B.** Promoter activity in lysates of cells that were grown in 0.09 mM of $CaCl_2$ and co-transfected with nLUC constructs that contain either 0, 1, 2, 3, 4 or 4.5 kb of the *SPINK7* promoter sequence and firefly vector. Promoter activity was determined by nLuc measurements relative to firefly measurements and normalized according to the promoter less nLUC measurements. Data are mean ± SEM.

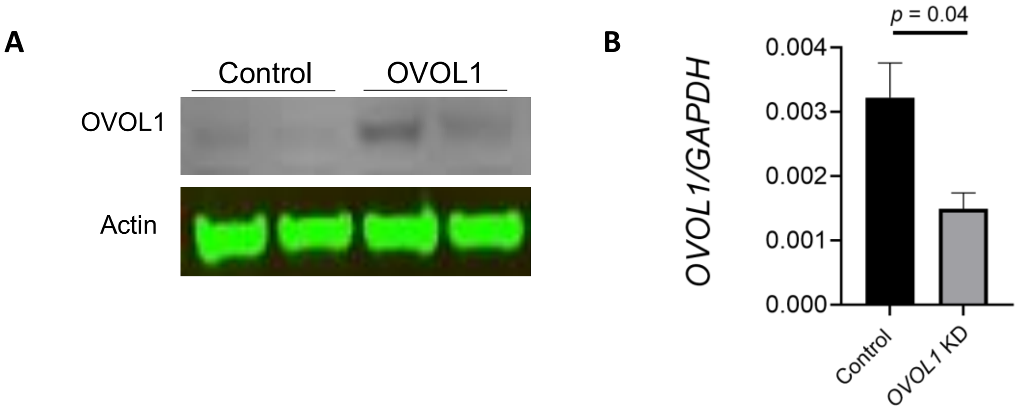

Supplementary Figure 2. Expression of OVOL1. A. Western blot analysis of OVOL1 in control or OVOL1-overexpressing EPC2 cells. β-actin was used as a loading control. **B.** Expression of *OVOL1* mRNA in non-silencing control cells (control) and *OVOL1*-silenced cells (*OVOL1* KD). Data are mean ± SEM.

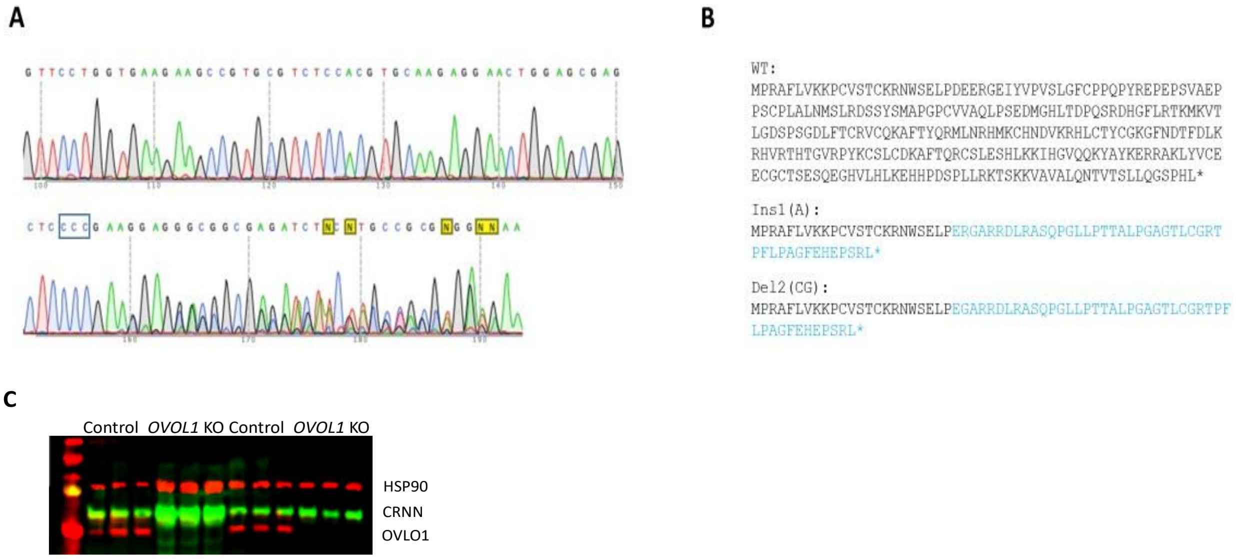

Supplementary Figure 3. Generation of *OVOL1* knockout EPC2 cells. A. A chromatogram depicting the genomic DNA sequence of EPC2 cells in the vicinity of the sequence targeted for CRISPR/Cas9-mediated editing. The box indicates the location of the PAM sequence. **B.** Prediction of the protein sequences of *OVOL1* knockout (KO) cells and control cells according to their genomic sequence. Black text indicates amino acids that match the wild type (WT) protein sequence. Blue text indicates amino acids that deviate from the WT protein sequence. C. Western blot analysis of *OVOL1* expression in 2 clones of *OVOL1* KO cells and 2 clones of control cells. HSP90 expression is presented as a reference gene and cornulin (CRNN) is presented as an example of differentiated marker of esophageal epithelium.

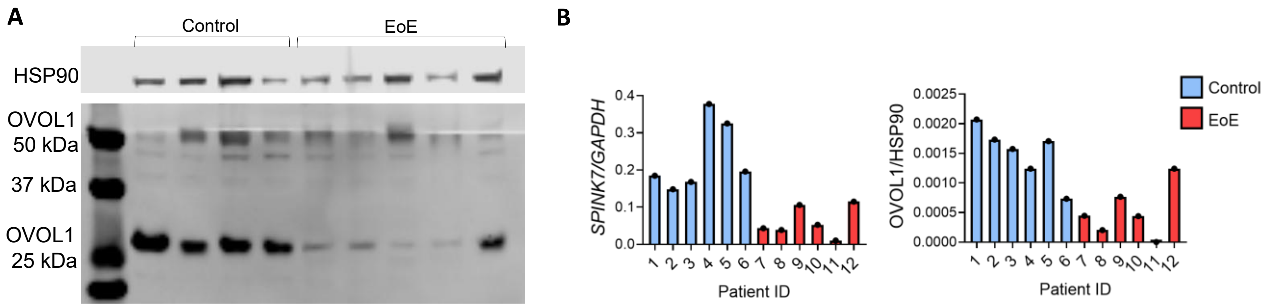

Supplementary Figure 4. OVOL1 expression in EoE and control esophageal biopsies. A. Unedited western blot image of OVOL1 expression in control and eosinophilic esophagitis (EoE) biopsies (as presented in figure 4D). **B.** Expression of OVOL1 protein and SPINK7 mRNA in 12 individual patients.

Supplementary Movie 1. Nuclear expression of OVOL1 after cellular differentiation. Reconstituted, three-dimensional confocal images of representative co-immunofluorescence staining of DSG1 (green), OVOL1 (pink), and DAPI (blue) staining in cells that were differentiated in air-liquid interface (ALI) culture.

Supplementary Movie 2. IL-13 inhibits OVOL1 nuclear localization after differentiation. Reconstituted, three-dimensional confocal images of representative co-immunofluorescence staining of DSG1 (green), OVOL1 (pink), and DAPI (blue) staining in cells that were differentiated in air-liquid interface (ALI) culture and were treated with IL-13 (100 ng/mL).

Supplementary Movie 3. IL-4 inhibits OVOL1 nuclear localization after differentiation. Reconstituted, three-dimensional confocal images of representative co-immunofluorescence staining of DSG1 (green), OVOL1 (pink), and DAPI (blue) staining in cells that were differentiated in air-liquid interface (ALI) culture and were treated with IL-4 (100 ng/mL).

Supplementary Table 1. List of candidate transcription factors (TFs) that may regulate SPINK7 expression. Bioinformatics analyses of TFs that are predicted to bind the *SPINK7* promoter sequence, TFs that are dysregulated in eosinophilic esophagitis (EoE) compared to control, TFs that are induced during epithelial differentiation, and TFs that are enriched in the esophagus (33).
