# Supplementary material for: IL-13 and calpain-14 suppress the expression of SPINK7 by regulating OVOL1 in eosinophilic esophagitis
Source: JCI Insight. 2026 Apr 28;11(12):e204687. doi: 10.1172/jci.insight.204687 (PMC13313498; doi:10.1172/jci.insight.204687)
Supplement: Unedited blot and gel images [file jciinsight-11-204687-s224.pdf]

HSP90

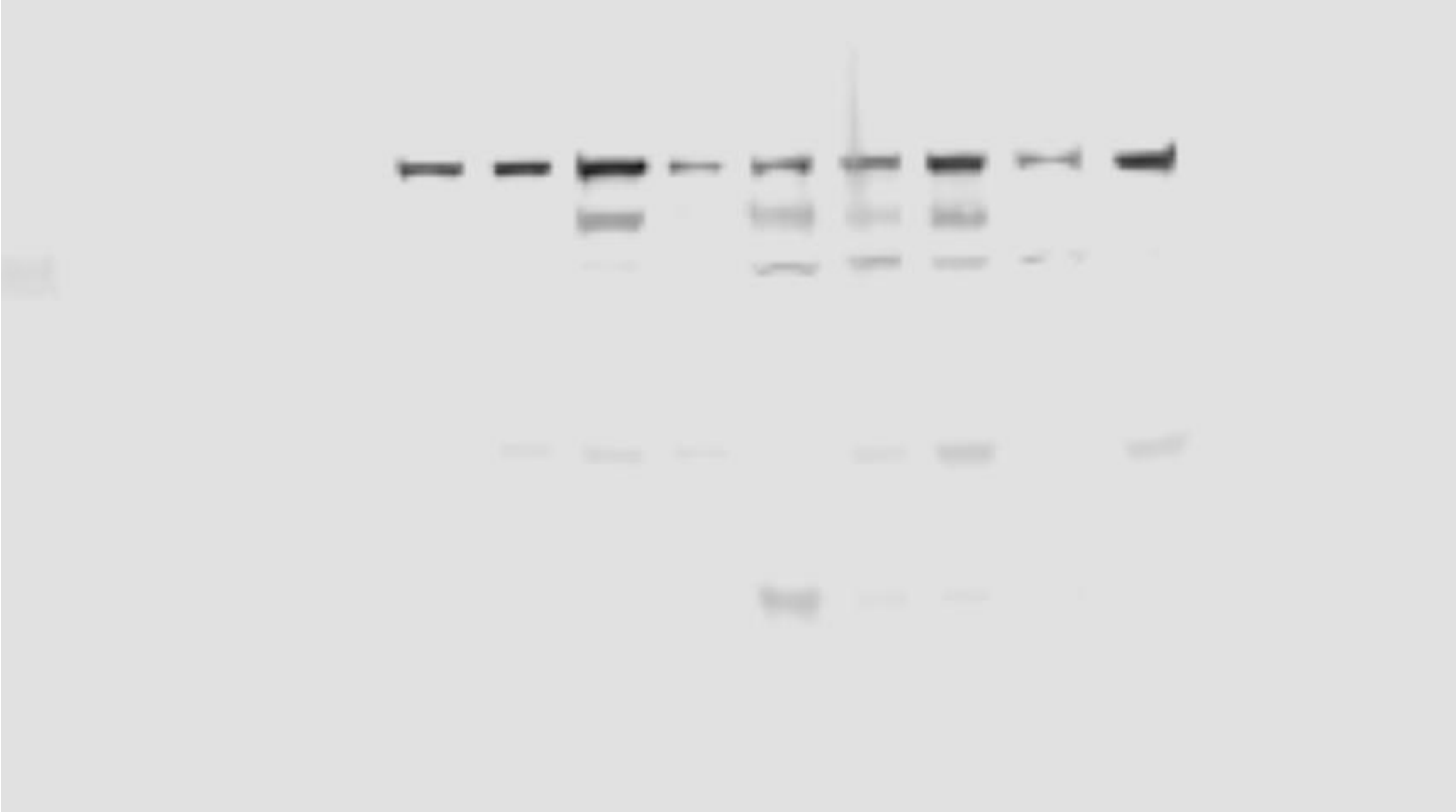

Membrane was scanned after probed for antiHSP90

Full unedited gel for Figure 4D

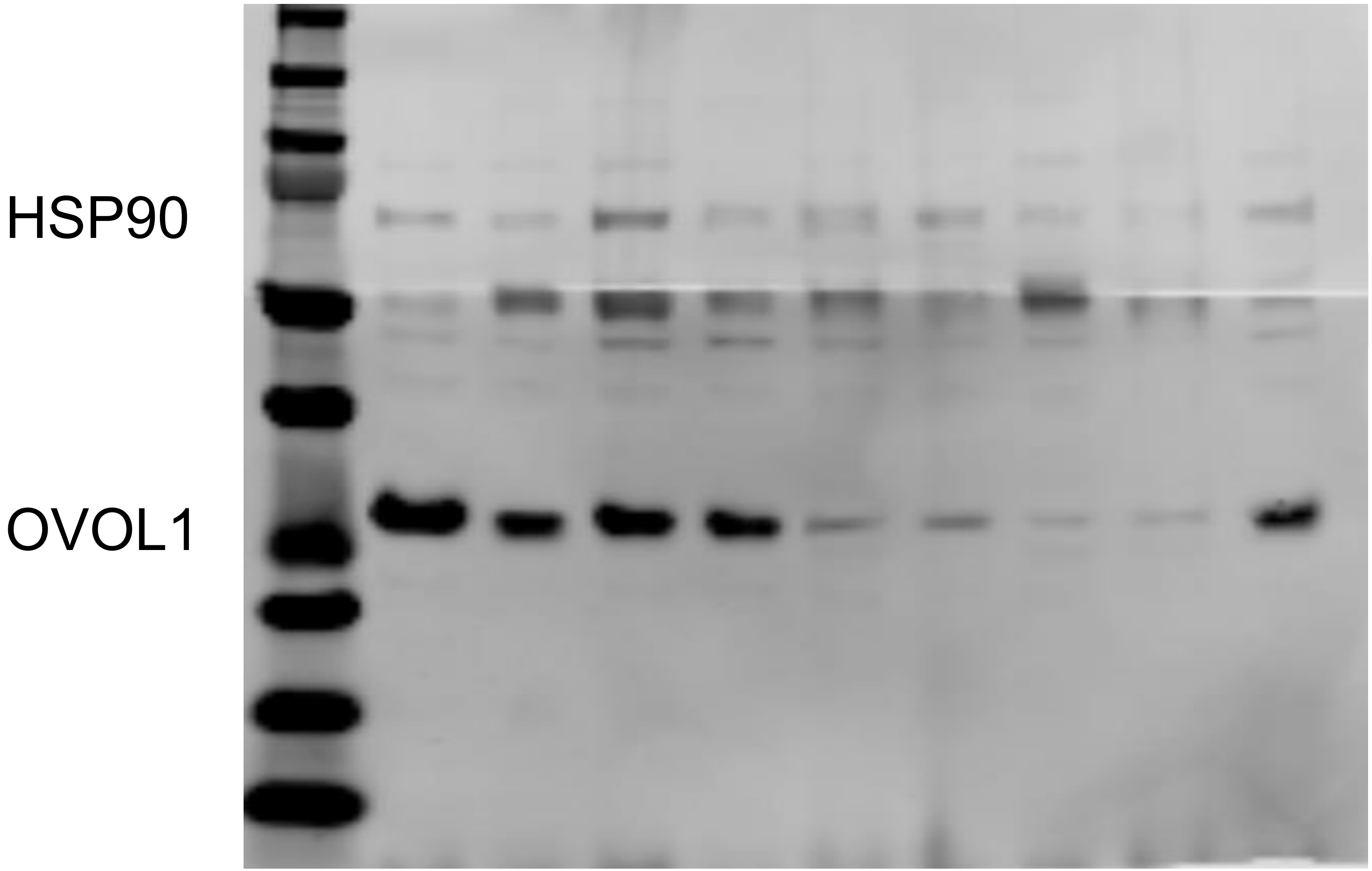

Membrane was cut, probed for antiOVOL1, and rescanned

Full unedited gel for Figure 4D

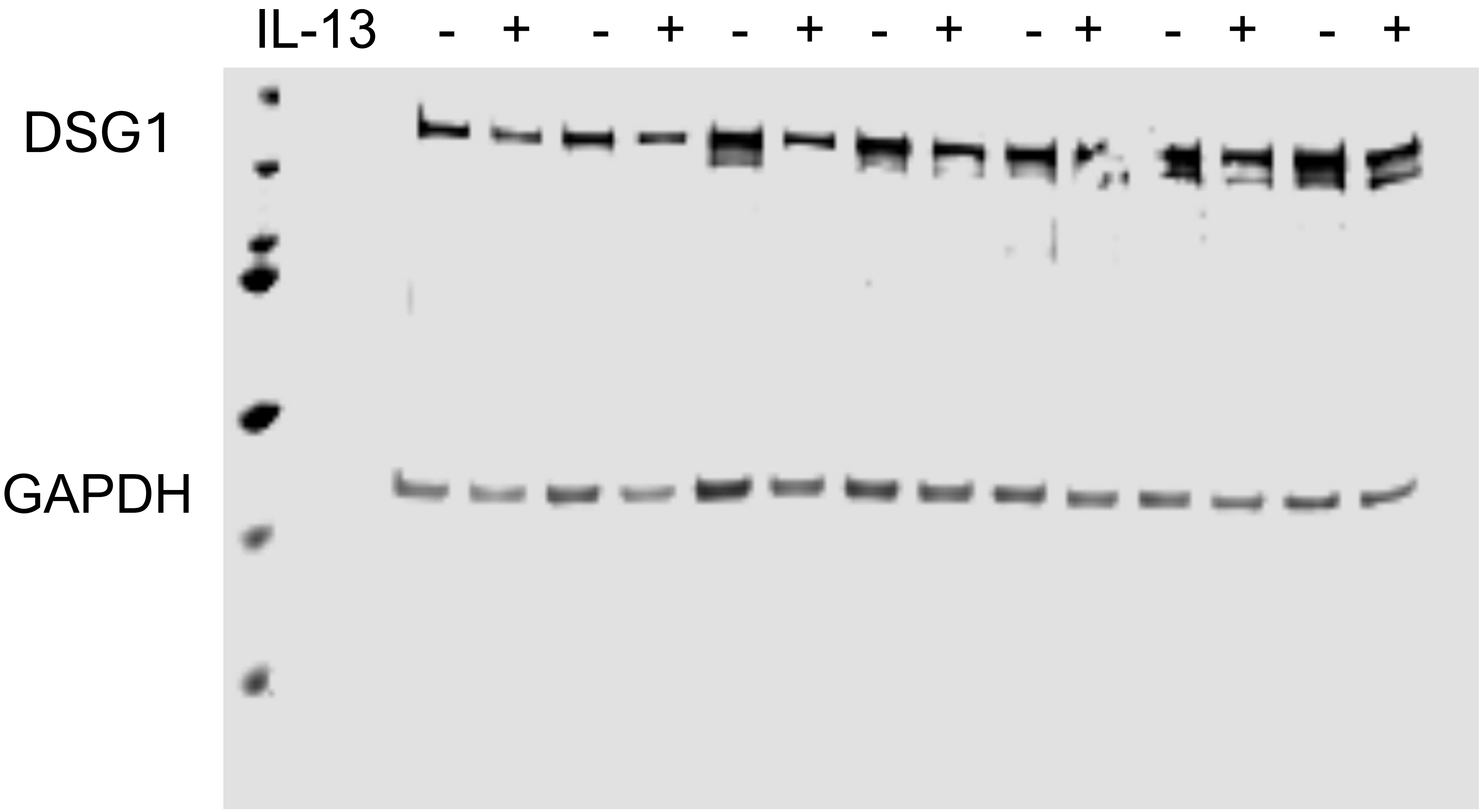

Full unedited gel for Figure 6A

OVOL1

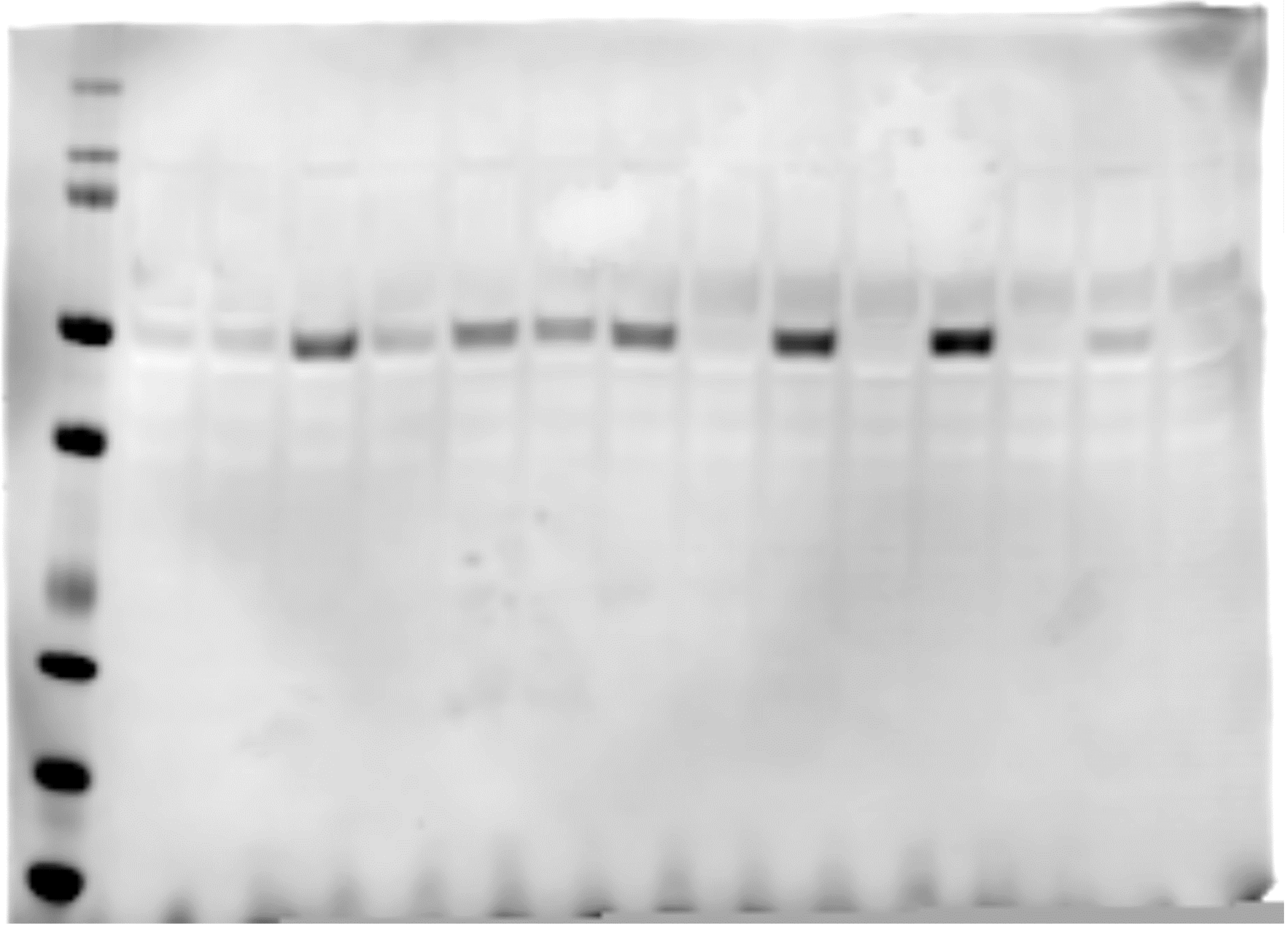

Full unedited gel for Figure 6A

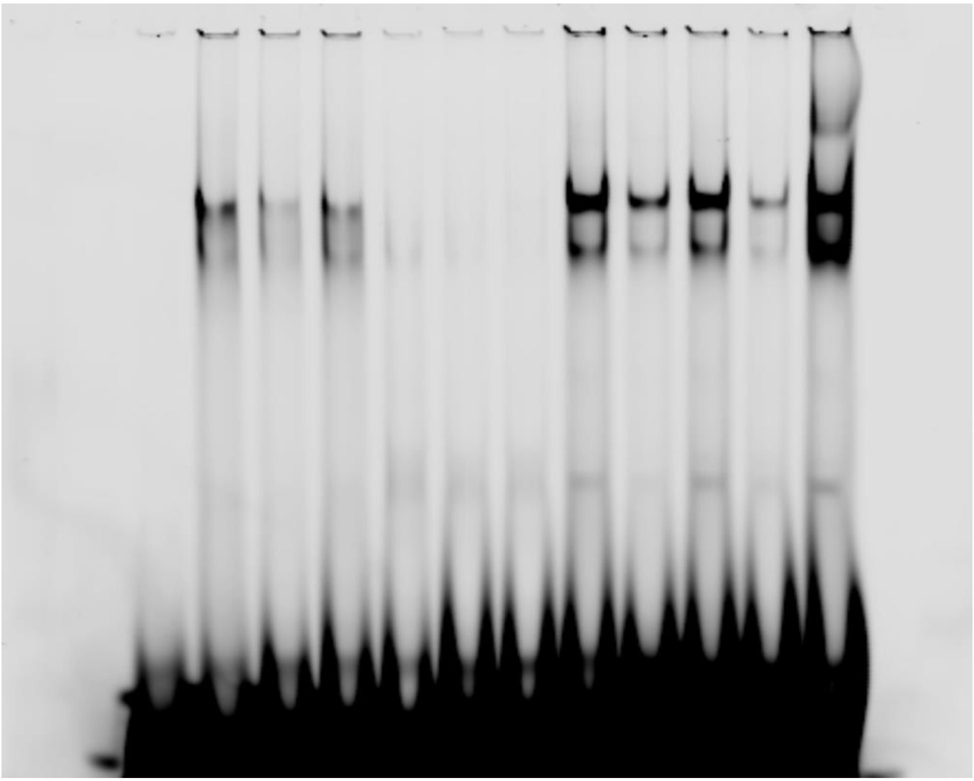

Full unedited gel for Figure 2D

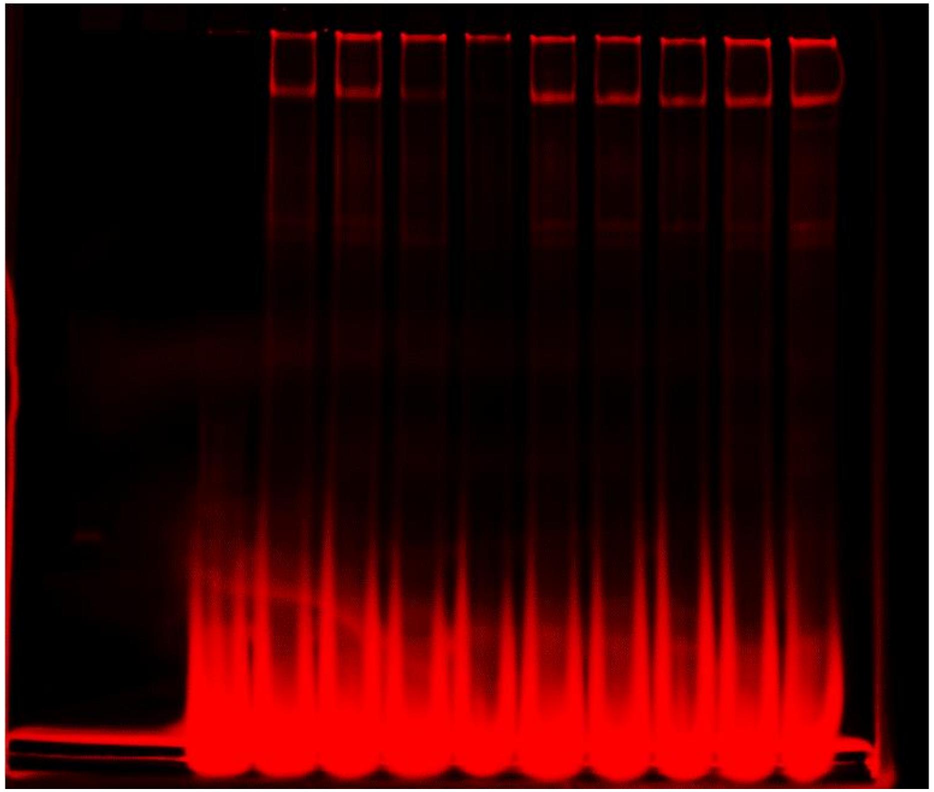

Full unedited gel for Figure 2E

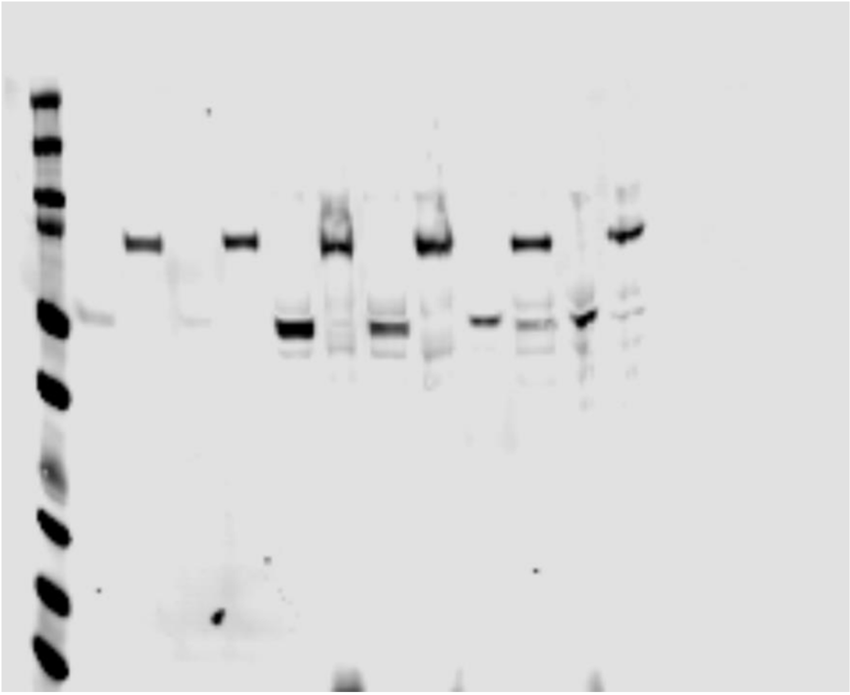

Full unedited gel for Figure 6C

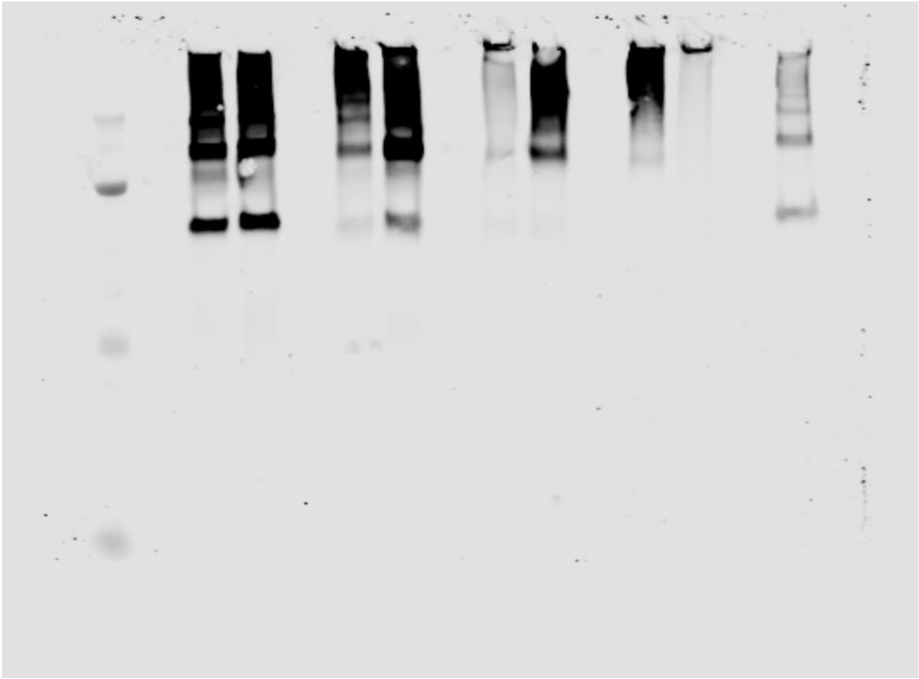

Full unedited gel for Figure 6G

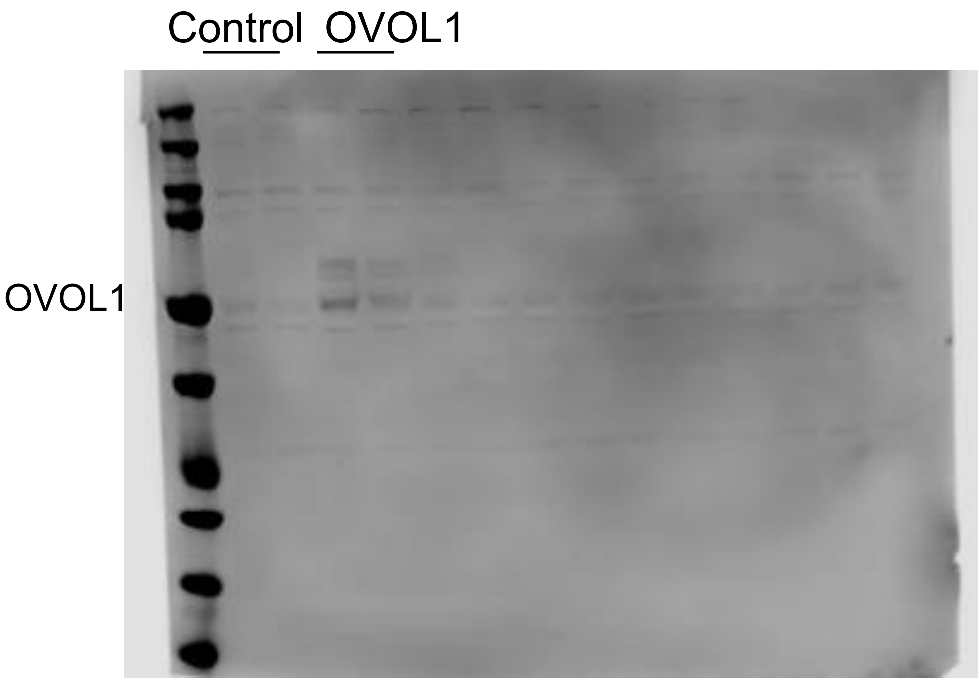

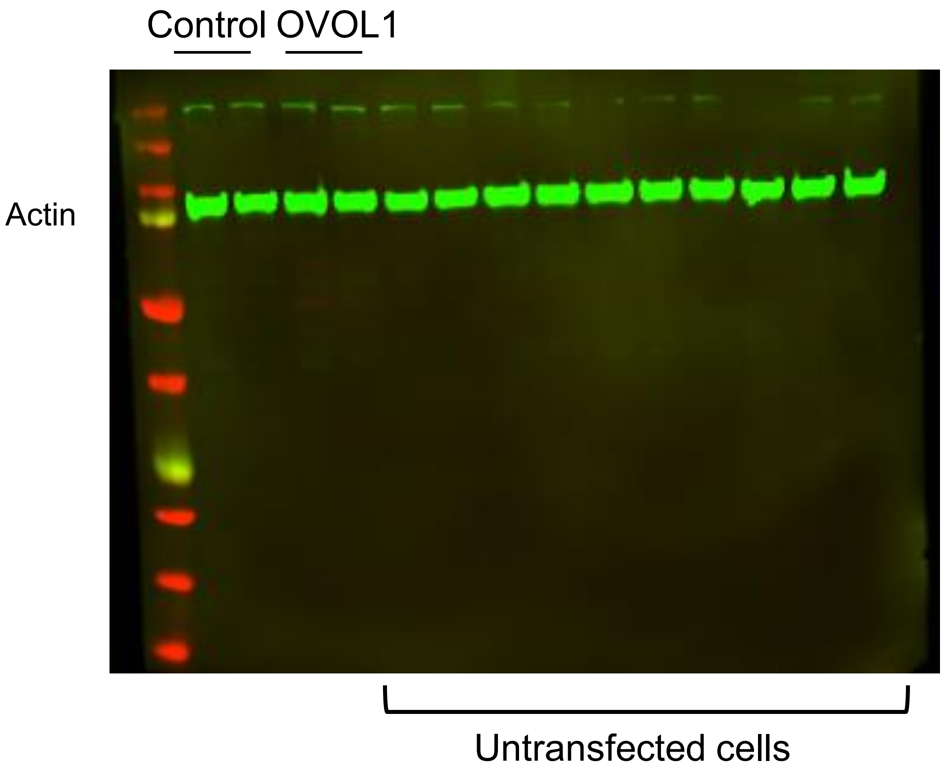

Full unedited gel for Figure SF2

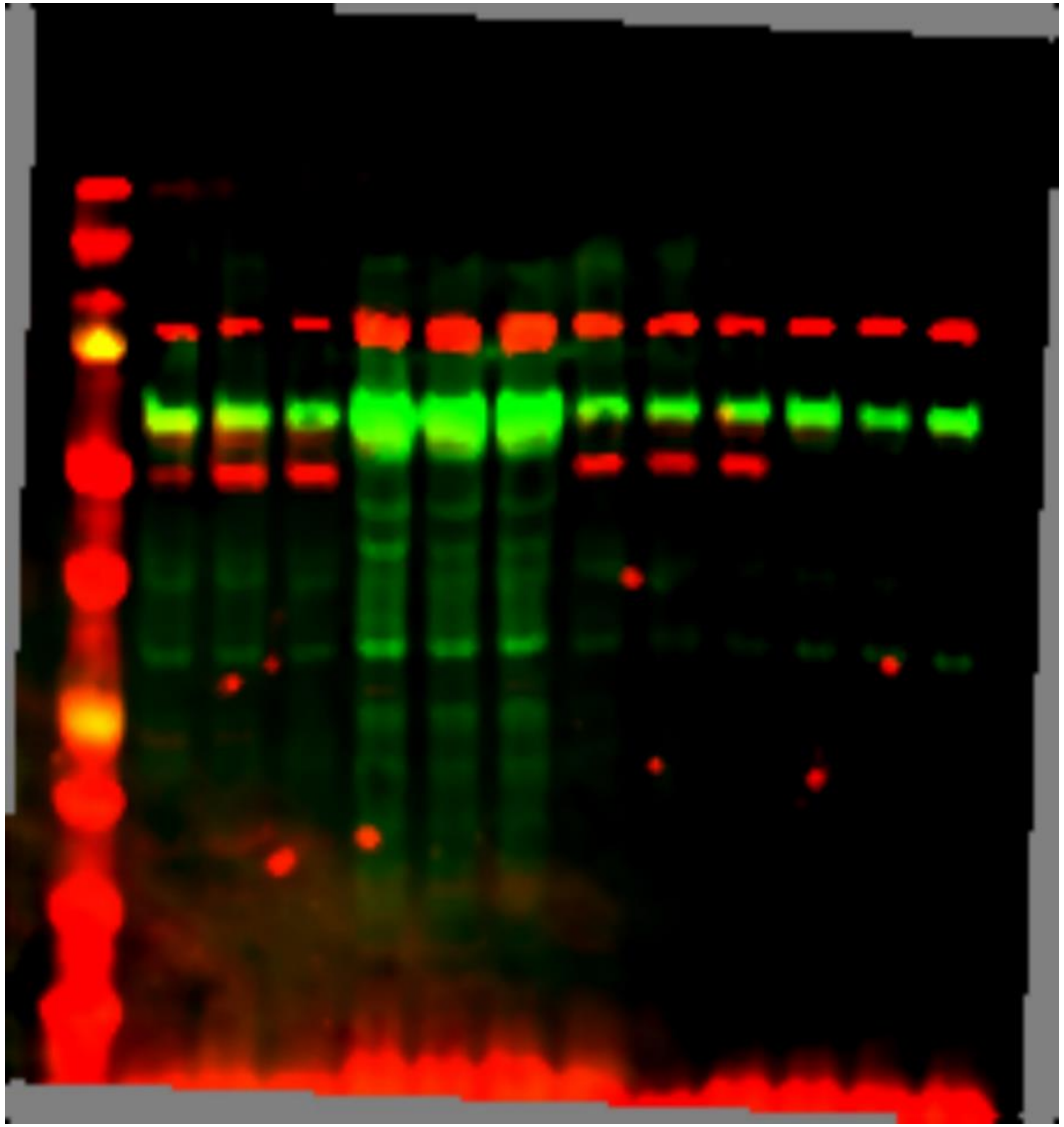

Full unedited gel for Figure SF3
